# Balanced Animal Nutrition for Better Feeds, Healthy Animals, Safe Food, and a Healthy Planet: A One Health Perspective—An Invited Review

**DOI:** 10.3390/ani16193138

**Published:** 2026-10-07

**Authors:** Eric N. Ponnampalam, Gauri Jairath, Rebecca Paul Weka, Tersur Theophilus Akpensuen, Hasitha Priyashantha, Charlotte Lauridsen, David Dazhia Lazarus, Gorakh Mal, Antonella Dalle Zotte, Sujatha Premaratne, Pam Dachung Luka

**Affiliations:** 1 School of Agriculture, Food and Ecosystem Sciences, Faculty of Science, The University of Melbourne, Parkville, VIC 3010, Australia; 2 Livestock Products Technology, ICAR-Indian Veterinary Research Institute, Regional Station, Palampur 176061, India; 3 Veterinary Extension Services Department, National Veterinary Research Institute, Vom 930103, Plateau, Nigeria; bekkyweka@gmail.com; 4 Sustainable Beef and Sheep Systems, Agri-Food and Biosciences Institute, Hillsborough BT9 6DR, UK; theo.akpensuen@afbini.gov.uk; 5 Department of Animal Science, Faculty of Agriculture, University of Jos, Jos 930001, Plateau, Nigeria; 6 Department of Animal Science, Faculty of Agriculture, University of Ruhuna, Kamburupitiya 81100, Sri Lanka; hasithap@agri.ruh.ac.lk; 7 Department of Animal and Veterinary Sciences, Aarhus University, DK-8830 Foulum, Denmark; charlotte.lauridsen@anivet.au.dk; 8 Infectious and Transboundary Animal Diseases, National Veterinary Research Institute, Vom 930103, Plateau, Nigeria; david.lazarus@nvri.gov.ng; 9 Animal Biochemistry, ICAR-Indian Veterinary Research Institute, Regional Station, Palampur 176061, India; gorakh.mal@icar.org.in; 10 Department of Animal Medicine, Production and Health (MAPS), University of Padova, Viale dell’Università 16, 35020 Legnaro, Padova, Italy; antonella.dallezotte@unipd.it; 11 Department of Animal Science, Faculty of Agriculture, University of Peradeniya, Peradeniya 20400, Sri Lanka; suep@pdn.ac.lk; 12 Biotechnology Centre, National Veterinary Research Institute, Vom 930103, Plateau, Nigeria; pamluka08@gmail.com

**Keywords:** animal-source foods, antimicrobial stewardship, circular bioeconomy, feed safety, livestock sustainability, One Health, precision nutrition, resilient livestock systems

## Abstract

Livestock production is increasingly expected to deliver not only higher output, but also better animal health and welfare, safer and more nutritious food, viable livelihoods, and lower environmental pressure. This invited review examines balanced animal nutrition within a One Health framework, linking feed resources with animal health, food quality and safety, community well-being, and environmental sustainability. Evidence from studies on improved forages, agro-industrial by-products, alternative feed resources, precision nutrition, feed safety, preventive healthcare, antimicrobial stewardship, manure management, and climate-resilient production was considered across the feed–animal–food–community–environment pathway. Nutritionally balanced and safe feeding strategies can support animal health and productivity, improve nutrient utilisation and modify selected quality characteristics of animal-derived foods. Their effects on disease occurrence, antimicrobial use, food safety and environmental outcomes depend on livestock species, physiological stage, dietary composition and production-system characteristics, as well as appropriate veterinary care and food-chain management. The review identifies differences among livestock species and production systems, as well as nutritional, economic and environmental trade-offs that influence the suitability of feeding strategies. Although evidence supports several individual relationships between animal nutrition, animal health, food quality and resource use, the complete sequence of downstream One Health outcomes has rarely been assessed within the same experimental or analytical framework. Integrated evaluation of these outcomes is therefore necessary to determine the wider benefits and limitations of nutritional interventions across livestock production systems.

## Simple Summary

Animal feeding affects not only productivity but also animal health, food quality, farm income and the environment. This review examines how better feeding practices can support healthier animals, safer and more nutritious foods, and more sustainable livestock production. It highlights the value of improved forages, locally available feed resources, food-processing by-products, safe alternative feeds and better feed management. These approaches may improve feed utilisation, animal health and product quality, although their wider food-safety and environmental benefits depend on production conditions and appropriate management. Overall, balanced animal nutrition can help create healthier animals, safer food, stronger farming communities and more sustainable food systems.

## 1. Introduction

Livestock production is no longer judged only by animal growth rate, reproductive performance, milk yield, egg output or carcass weight. It is now increasingly evaluated by a wider set of questions: how efficiently animals use feed, how safely they are managed, how much pressure production places on land and water, and whether the foods produced genuinely contribute to human nutrition without adding public-health issues or environmental risks. These concerns are not separate. Feed scarcity, high feed costs, climate stress, disease outbreaks, antimicrobial resistance and environmental degradation often act together, especially in systems where farmers have limited access to quality feed, veterinary support and stable markets. Building resilient livestock systems therefore requires approaches that maintain productivity while protecting animal, human and environmental health [1,2].

The One Health approach is useful in this context because livestock systems sit directly at the interface of animals, people, plants and ecosystems. The Quadripartite One Health Joint Plan of Action calls for coordinated action across these sectors to prevent, predict, detect and respond to health threats at their points of connection [3]. In livestock production, these points of connection are visible at every stage: crops and by-products become feed; feed influences animal health and product quality; animal-source foods affect human nutrition; and manure, emissions, land use and waste streams influence environmental quality. Thus, a review on balanced animal nutrition must move beyond nutrient supply alone and examine the wider feed–animal–food–community–environment integrated pathway [4,5].

Feed is one of the most practical entry points in this pathway. It is usually the largest recurring input in livestock production and strongly influences productivity, immunity, welfare, reproduction, environmental efficiency and the quality of meat, milk and eggs. The idea of “One Nutrition” is particularly relevant here because nutrients move across crop, animal and human food systems rather than remaining confined to one sector [6]. A balanced diet for livestock should therefore be understood not only as a ration that meets requirements for protein, energy, minerals and vitamins, but also as a tool for improving resource use, reducing avoidable losses and supporting safer food-production systems.

Poor feeding has consequences that extend well beyond reduced performance. Inadequate energy or protein supply, mineral and vitamin deficiencies, poor-quality roughage, contaminated ingredients and seasonal feed gaps can weaken immune competence, impair reproduction, reduce performance or product yield, and increase the likelihood of disease and maintenance costs [6,7]. On the other hand, well-planned nutritional strategies can improve lifetime productivity, herd efficiency, welfare, product quality and farm profitability while reducing nutrient wastage and environmental pressure. This makes balanced nutrition a preventive and strategic component of livestock health, not merely a production expense.

Feed quality also determines the nutritional value and safety of the foods derived from animals. Diets can influence the fatty acid profile, antioxidant status, micronutrient content, oxidative stability and sensory quality of meat, milk and eggs. Precision feeding, functional ingredients, biotechnology-supported feed improvement and other nutritional innovations may help enrich animal-derived foods with beneficial components such as omega-3 fatty acids, antioxidants and bioactive compounds when used appropriately [8]. At the same time, unsafe or poorly controlled feeds may carry mycotoxins, pesticide residues, heavy metals, pathogenic microorganisms or other hazards into the animal and, in some cases, into the food chain. Feed safety is therefore an early and essential part of food safety and ultimately human health and well-being.

The sustainability of livestock production will depend heavily on how feeding strategies and production systems are designed. Improved forages, better pasture management, local feed resources, crop residues, agro-industrial by-products, circular bioeconomy approaches, precision nutrition and feed-safety systems can all contribute to more resilient livestock production when adapted to local conditions. Their value, however, should not be assumed simply because they are local, novel or circular. They must be nutritionally adequate, economically feasible, safe for animals and consumers, and compatible with environmental goals [1,2,9].

Previous reviews have examined the use of forages, supplements and agricultural by-products in relation to livestock productivity and meat quality [5], the connections among crop production, animal nutrition and human nutrition [6], and the implementation of One Health across different sectors [4]. Building on these perspectives, the present review uses balanced animal nutrition as its point of entry to examine how feeding strategies influence animal health, the quality and safety of animal-derived foods, resource use and their wider implications for communities and the environment. Its specific contribution is to examine these relationships within a common feed–animal–food–community–environment framework, distinguishing directly measured outcomes from broader One Health benefits that depend on additional management, economic and environmental factors.

**Figure 1:**
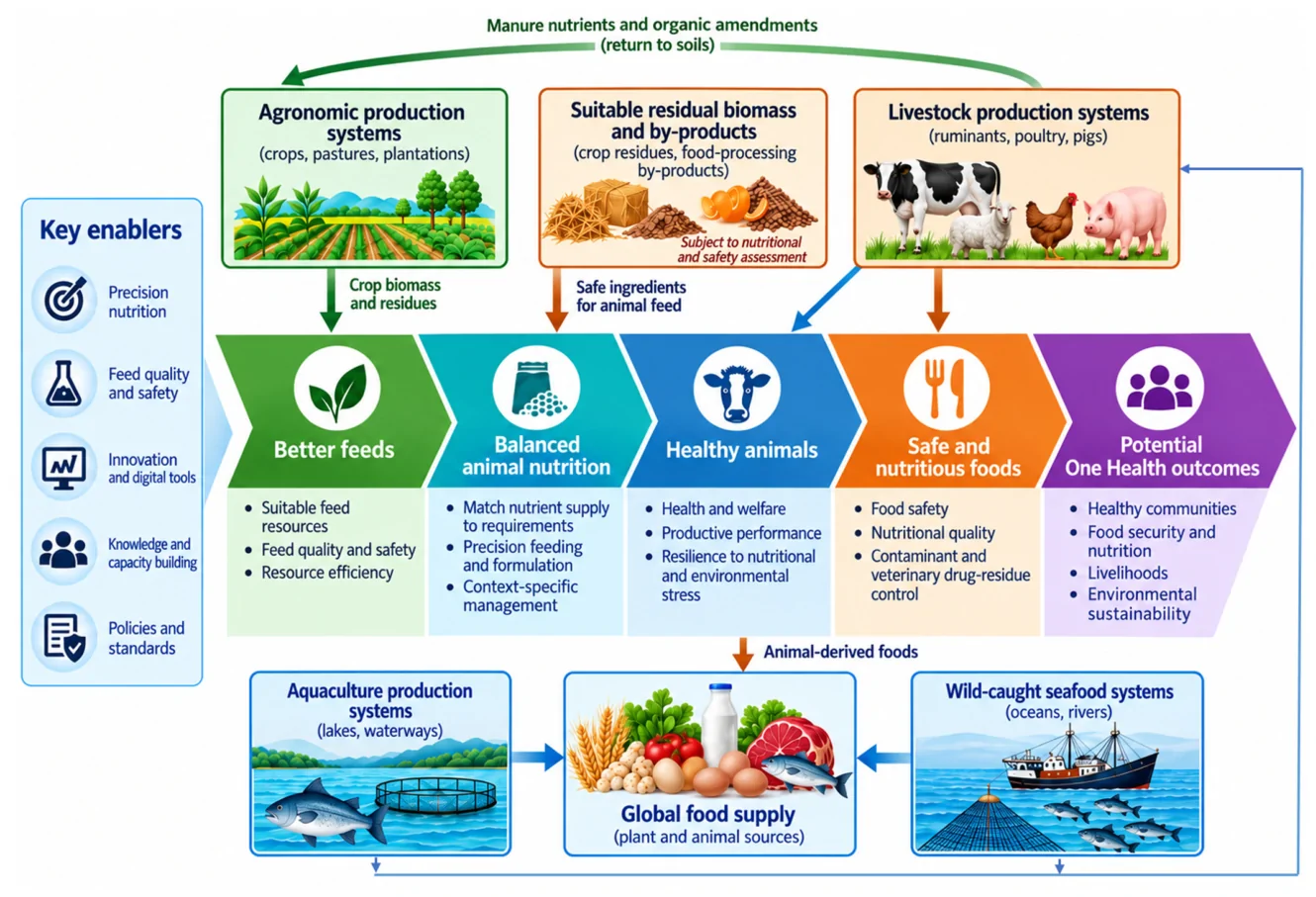
Integrated One Health framework linking balanced animal nutrition with animal health, food quality and safety, community well-being, and environmental sustainability through interconnected food-production systems and circular resource flows [9]. Images within the figure were generated using Microsoft Copilot AI version 2.2.

The review focuses on forage management, alternative feed resources, precision nutrition and feed safety across livestock species and production systems. Veterinary care, antimicrobial stewardship, animal welfare, food processing, livelihoods and environmental management are considered wherever they influence the implementation or outcomes of nutritional interventions, rather than as independent areas of review. Particular attention is given to differences among livestock species and production systems, nutritional and environmental trade-offs, and practical constraints affecting the adoption of feeding strategies. The proposed pathway provides a framework for examining these relationships and identifying where evidence of downstream One Health outcomes remains limited (Figure 1).

## 2. Materials and Methods

This invited narrative review involved a structured search of Scopus, Web of Science, PubMed and Google Scholar to identify relevant publications available up to August 2026. Search terms were used individually and in combination and covered animal nutrition, feed resources, forages, agro-industrial by-products, precision nutrition, feed safety, animal health and welfare, antimicrobial resistance and stewardship, animal-source foods, food safety, livelihoods, circular bioeconomy, climate resilience, livestock sustainability and One Health. Boolean operators were used where appropriate, and reference lists of relevant articles, reviews and reports were screened to identify additional sources. The search was organised around the main components of the proposed feed–animal–food–community–environment pathway, with related terms combined to identify publications examining nutritional interventions and their animal-health, food-quality, environmental and socioeconomic outcomes. Peer-reviewed research and review articles were prioritised, together with authoritative reports and guidance documents from recognised international organisations; earlier publications were retained, where they provided established concepts or evidence of continuing relevance.

Publications were considered for inclusion if they examined the nutritional suitability of feed resources or the effects of feeding strategies on animal performance, health, product quality and safety, resource use, or environmental outcomes. Studies addressing veterinary care, antimicrobial stewardship, food processing, livelihoods and environmental management were also considered wherever they contributed to understanding the wider implications of nutritional interventions within the One Health framework. Publications without a relevant connection to animal nutrition or the proposed feed–animal–food–community–environment pathway were excluded. The relevance of identified publications was assessed from their titles, abstracts and, where necessary, full texts, with sources retained according to their contribution to the topics addressed in this review.

Because of the diversity of livestock species, production systems, interventions and reported outcomes, the evidence was synthesised qualitatively rather than by meta-analysis. The synthesis considered differences in livestock species, feed resources, dietary inclusion levels, experimental conditions and measured outcomes. Where available, studies reporting favourable, neutral or unfavourable responses were considered to identify differences in the effects of nutritional interventions across production conditions. Primary research articles were used to support specific experimental findings, while review articles and authoritative reports provided broader scientific and policy context. Findings were organised according to the main components of the proposed pathway and compared in relation to the measured outcomes, experimental conditions and characteristics of the livestock production systems. The selected studies were interpreted according to their measured outcomes, without assuming that improvements in animal performance or product quality necessarily translate into wider human-health or environmental benefits. As this was a narrative review, the literature search was not intended to provide an exhaustive identification of all eligible studies, and no formal risk-of-bias assessment was undertaken. The possibility of selection bias therefore remains a limitation of the review.

A total of 168 publications, including original research articles, reviews and authoritative reports, were included in the review. Microsoft Copilot version 2.2 was used to generate illustrative elements incorporated into the figures.

## 3. Better Feeds: Formulation of Sustainable, Safe and Resilient Animal Diets

**Figure 2:**
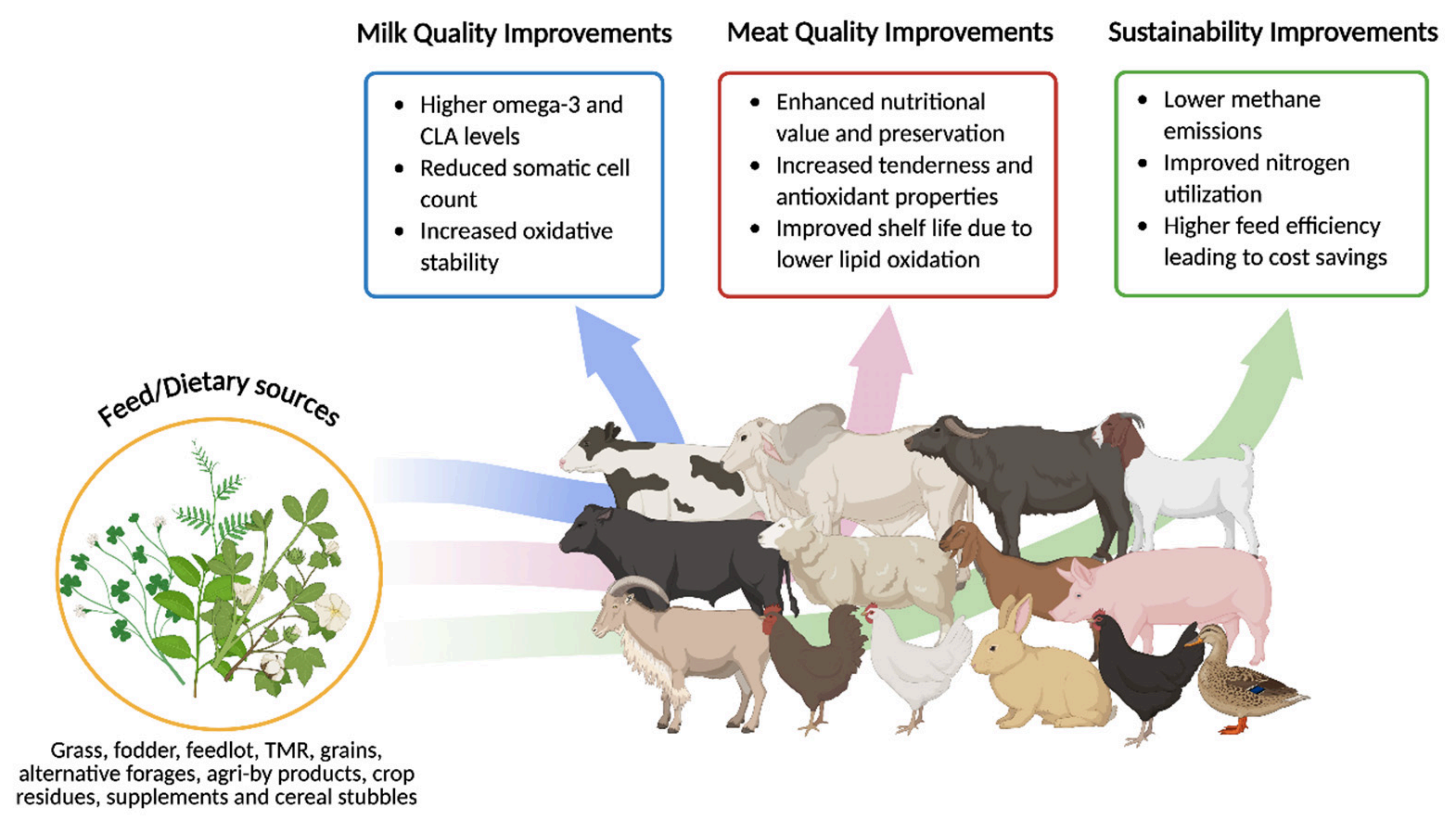
Influence of feed and dietary sources on milk quality, meat quality and sustainability outcomes across livestock species. Modified from Ponnampalam et al. [11].

The nutritional quality of animal-derived foods is strongly shaped by on-farm factors. Species, breed, age, physiological status, diet, health status, production system and environmental stress can influence the composition and quality of meat, milk and eggs. The fatty acid, antioxidant, vitamin and trace-element composition of the diet can alter oxidative and colour stability and sensory properties of animal products [8,10]. An appropriate and strategic use of balanced functional feed ingredients can enhance product quality (meat, milk or eggs), economic stability and social acceptance, as described in Figure 2.

Nutritional enhancement should not be considered an automatic outcome. It depends on the dose, duration and quality of the ingredients, as well as their bioavailability and bioactivity, the product matrix and storage conditions, and host factors such as animal species, rumen biohydrogenation in ruminants, age or physiological status of the animal, and the gut microbiome [12]. Insufficient antioxidant protection and inadequate packaging and storage may also lead to lipid oxidation, thereby reducing the nutritional value of products enriched with unsaturated fatty acids. Therefore, a nutritional approach before harvest needs to be integrated with post-harvest quality control of the nutritional profile to prevent losses due to oxidation, microbial spoilage or poor handling [10].

Food quality is also influenced by animal health. Disease, inflammation, parasitism, metabolic stress and poor welfare may reduce milk yield and composition and affect growth, carcass quality and susceptibility to contamination during production or processing [13,14]. Stress prior to slaughter may affect meat quality through changes in glycogen reserves and abnormal post-mortem pH [15,16], while mastitis and systemic infections may affect milk quality and safety. Similarly, heat-stressed birds frequently exhibit altered post-mortem muscle metabolism, resulting in abnormal pH decline, poorer water-holding capacity, colour defects and increased oxidative damage, ultimately reducing both technological and nutritional quality of poultry meat [17]. Likewise, in pigs, pre-slaughter stress associated with transport, mixing of unfamiliar animals and handling may accelerate muscle glycolysis, increasing the incidence of pale, soft and exudative (PSE) meat, thereby reducing water-holding capacity, processing suitability and consumer acceptance [18]. Hence, food quality is not determined by processing technology alone; it also begins with healthy animals, good animal welfare and preventive farm management.

### 3.1. Foundations and Forage-Based Strategies for Better Feed

Feed formulation must account for nutrient requirements, feed availability, ingredient quality and cost, particularly where seasonal feed scarcity and climate variability constrain livestock production [1,6,19]. Forages provide a major source of nutrients in ruminant diets and contribute to soil conservation and nutrient cycling when appropriately managed [20,21]. However, seasonal fluctuations in forage quantity and quality can limit animal performance, particularly in tropical and subtropical production systems [22,23]. Improved pastures, fodder conservation, fodder intensification and dual-purpose food–feed crops can help address seasonal feed deficits, although their suitability depends on local climatic conditions, land availability and production requirements [1,21].

Leguminous forages and fodder trees, including *Leucaena leucocephala*, *Gliricidia sepium*, *Desmodium* spp. and *Stylosanthes* spp., can contribute to dietary protein supply and soil nitrogen enrichment through biological nitrogen fixation [24,25]. Their nutritional suitability, however, depends on species, maturity, chemical composition and dietary inclusion level. Silvopastoral systems that integrate trees, forages and livestock can improve feed availability, provide shade, and support nutrient cycling and carbon storage, although these outcomes depend on vegetation composition, grazing management and local environmental conditions [20,26].

In pigs, poultry, rabbits and quails, alternative feed resources, including oilseed cakes, agro-industrial by-products and insect-derived proteins, can partially replace conventional feed ingredients when appropriately formulated [27,28,29,30]. However, their application differs from that of forage-based strategies in ruminants because of differences in digestive physiology, nutrient requirements and the capacity to utilise fibrous feed resources. Their effects on nutrient utilisation, productive performance and product quality also vary with ingredient composition and inclusion level. The selection of feeding strategies should therefore consider the nutritional requirements of the target species, local feed availability, processing requirements, and the economic and environmental implications of replacing conventional ingredients.

### 3.2. Precision Nutrition, Feed Efficiency and Animal Resilience

Precision nutrition aims to match nutrient supply more closely with the requirements of individual animals or groups, accounting for differences in body weight, physiological stage, feed intake and productive performance [31]. Its application involves integrating feed-composition data, animal-level measurements, nutrient-requirement models and systems capable of adjusting feed allocation or dietary composition. Electronic identification, automated weighing and feeding systems, and sensors monitoring feed intake, rumination and activity can provide information for these decisions [32]. In dairy cattle, sensor-based monitoring has demonstrated changes in rumination, eating and locomotor behaviour under heat stress, indicating that feeding-related measurements must be interpreted in relation to environmental conditions and animal health [33]. Individual milk-yield, feed-intake and body-weight records can also inform adjustments to concentrate allocation through automated feeding systems, although the effects on milk production and nutrient-use efficiency depend on the feeding strategy and the accuracy of nutrient-requirement estimates [34]. Subsequent measurements can be used to reassess nutrient requirements and modify the feeding programme. The usefulness of precision feeding therefore depends on the accuracy of animal-level measurements, nutrient-requirement models and feeding algorithms, together with appropriate veterinary and management support.

Near-infrared spectroscopy (NIRS) can complement animal-level monitoring by providing rapid estimates of feed composition. Portable and laboratory-based NIRS instruments can estimate moisture, crude protein and fibre contents in forages and feed ingredients when suitable calibration models are available [35]. For example, analysing successive batches of silage or agro-industrial by-products can help identify variation in nutrient composition and support adjustments to dietary formulation. Combining these measurements with feed-intake and animal-performance records can improve the information available for nutrient allocation. However, NIRS prediction accuracy depends on calibration quality, sample characteristics and the range of feed ingredients represented in the calibration dataset. Models developed for conventional feed ingredients may not perform adequately when applied to heterogeneous crop residues or novel feed resources. Furthermore, routine NIRS measurements of nutrient composition cannot establish microbiological or chemical feed safety. Such applications require separately validated analytical methods and appropriate confirmatory testing.

Experimental studies illustrate both the potential and limitations of precision feeding. In growing-finishing pigs, daily adjustment of dietary composition according to individual nutrient requirements reduced standardised ileal digestible lysine intake by 27%, estimated nitrogen excretion by 22% and estimated phosphorus excretion by 27% compared with conventional three-phase feeding, without significant differences in growth performance or carcass composition [36]. In dairy cattle, individual adjustment of concentrate supplementation according to estimated nutrient requirements reduced urinary nitrogen excretion without significantly affecting short-term energy-corrected milk yield [37]. However, methane emissions from stored manure increased in the precision-fed group, indicating that reduced nitrogen excretion did not necessarily translate into lower emissions across all measured environmental outcomes. A more recent dairy-cattle study reported that variable concentrate allocation using an automated feeding system reduced milk yield and feed efficiency compared with conventional total mixed ration feeding, whereas energy-corrected milk yield was not significantly affected [34]. These findings indicate that more frequent or individualised nutrient allocation does not necessarily improve productive performance or overall environmental outcomes. Its effects depend on the accuracy of nutrient-requirement estimates, feeding-system design, dietary formulation and subsequent management of nutrient losses.

The wider application of precision nutrition will depend on integrating feed analysis, animal monitoring and automated nutrient allocation into feeding systems that are accurate, economically feasible and adaptable to different production conditions. This requires validation of sensors and nutrient-requirement models across livestock species, breeds, physiological stages and locally available feed resources. The effects of precision feeding should also be assessed beyond feed efficiency and productivity, incorporating animal health and welfare, nutrient excretion, manure-related emissions, product quality and food-safety indicators where appropriate. Such assessments are necessary to determine whether improvements in nutrient allocation translate into broader One Health benefits. Equipment costs, technical support, data compatibility and access to reliable feed analysis remain practical constraints, particularly in smallholder systems [31,32]. Where automated systems are not economically feasible, periodic feed analysis, body-condition scoring, production records and group feeding according to physiological stage may provide more accessible alternatives. The application of precision nutrition should therefore be guided by measurable nutritional, animal-health, economic and environmental outcomes, rather than the availability of monitoring technologies alone.

Crop residues and agro-industrial by-products are important resources for reducing feed costs, improving biomass utilisation and strengthening circular crop–livestock systems. Cereal straws and stovers, sugarcane tops, oilseed cakes, brewers’ and distillers’ grains, molasses, fruit and vegetable processing residues, former food products, and other food-industry by-products can supply valuable nutrients when properly evaluated and incorporated into balanced diets [38,39,40]. However, their suitability is limited by variable composition, poor digestibility, bulkiness, anti-nutritional factors and possible contamination [41]. These limitations do not preclude their use but highlight the need for appropriate processing technologies and nutritional evaluation to improve their feeding value while ensuring animal health and feed safety. Thus, circularity should not be pursued at the expense of animal health or feed safety; nutritional analysis and risk assessment must precede their use [42].

Growing evidence indicates that several agro-industrial by-products can be incorporated into livestock diets when appropriately processed and nutritionally balanced (Table 1). In dairy cattle, citrus pulp increased selected antioxidant-associated components of milk, while dried grape pomace maintained most milk-composition parameters [43,44]. Dried apple pomace maintained milk yield and most milk-composition traits, although fat-corrected milk yield and milk fat yield decreased with increasing inclusion levels [45]. Olive pomace modified the fatty acid composition and aromatic profile of milk and cheese [46]. Oilseed cakes have also been evaluated as alternative protein sources in ruminant diets, with responses varying according to oilseed source, processing and dietary inclusion level [47,48,49]. Hempseed cake showed inclusion-dependent effects on milk production and composition, with higher inclusion levels reducing crude protein-use efficiency [50].

Among agro-industrial by-products, cold-pressed oilseed cakes have been evaluated as alternative protein and energy sources in pig diets. Studies on canola, camelina, rapeseed, linseed and hempseed cakes have reported differences in nutrient and amino acid digestibility according to oilseed source and processing conditions [51,52,53,54,55]. These differences are relevant to dietary formulation because the nutrient content of an ingredient does not necessarily reflect its digestible nutrient supply. In fattening pigs, dietary inclusion of camelina oil cake at 12% maintained productive performance while modifying selected metabolic and immune-related responses [56]. The use of hydrothermally processed soybean- and rapeseed-based diets also influenced selected performance, carcass and backfat fatty acid characteristics [57]. These findings indicate that oilseed cakes should be evaluated according to their digestible nutrient supply, processing characteristics and effects on animal performance rather than treated as interchangeable protein sources.

Oilseed cakes have also been evaluated as partial substitutes for conventional protein sources in poultry diets. In laying hens, hempseed meal at inclusion levels up to 20% did not significantly affect productive performance or egg quality, while increasing selected polyunsaturated fatty acids in eggs [58]. In contrast, higher inclusion levels of rapeseed, linseed and hempseed cakes adversely affected selected laying-performance indices, with responses differing among the oilseed sources [59]. In broilers, dietary camelina cake increased n-3 polyunsaturated fatty acid deposition in meat and reduced the n-6/n-3 ratio without adversely affecting growth performance at the tested inclusion level [60]. In broilers receiving camelina meal at 3% of the diet, chromium supplementation increased breast meat protein, Zn and Fe content, and reduced crude fat and lipid oxidation, while camelina meal increased n-3 PUFA deposition without significantly affecting growth performance [61]. In laying hens, camelina oil and cake increased n-3 polyunsaturated fatty acid deposition in egg yolk, although responses in laying performance, egg quality and sensory characteristics differed between treatments [62].

Recent studies on hemp-derived feed resources provide further evidence of the differences between nutritional and broader sustainability outcomes. In medium-growing organic broilers, dietary inclusion of 15% and 30% feed-grade hempseed maintained carcass yield and most meat-quality traits while increasing the proportion of polyunsaturated fatty acids in breast meat [79]. In organic laying hens, 30% locally produced hemp cake maintained laying performance and measured behavioural characteristics while increasing egg yolk omega-3 fatty acids and reducing the n-6/n-3 ratio. However, feed-related production costs and estimated greenhouse gas impacts increased compared with the control diet [80]. These findings illustrate that maintaining productive performance and modifying product composition do not necessarily establish economic or environmental benefits. The applicability of these feeding strategies depends on ingredient characteristics, dietary formulation, production conditions and the boundaries of the environmental assessment.

Oilseed cakes have also been evaluated in quails and rabbits, with responses varying according to the feed ingredient, inclusion level and physiological stage. In growing and laying quails, dietary inclusion of 15% camelina cake generally preserved carcass and egg quality, although productive responses were influenced by the camelina genotype and the relatively high inclusion level, resulting in reduced growth performance or egg production [63,64]. Likewise, in rabbits, moderate dietary inclusion of camelina (5%) or hempseed cake (up to 10%) did not adversely affect health status or productive performance, whereas higher camelina cake inclusion (10%) negatively affected nutrient digestibility and growth performance, indicating the importance of optimising dietary inclusion levels according to the nutritional characteristics of each co-product [65,66].

Biological processing, particularly microbial and solid-state fermentation, can improve the digestibility, nutrient availability and protein content of low-quality residues while reducing some anti-nutritional compounds [40,81]. Nevertheless, processing outcomes depend on substrate quality, microbial culture, moisture, temperature, hygiene and cost. For example, self-fermentation of apple pomace, spent mushroom compost and wheat straw has been shown to produce a nutritionally viable maize-replacing feed resource, with favourable effects on ruminal fermentation, feed conversion and oxidative status in goats [82]. Therefore, fermentation should be treated as a targeted upgrading strategy rather than a universal solution. Its practical adoption requires standardised methods that produce safe, consistent, affordable and farmer-acceptable feeds.

Novel resources including insect meals, algae, seaweed, microbial proteins, aquatic biomass and locally available plants may further diversify feed supplies and reduce dependence on human-edible grains and imported protein meals [83,84]. Insect meals derived from *Tenebrio molitor* and *Hermetia illucens* have been evaluated as partial replacements for conventional protein sources in broiler chickens and quails [67,68,69,70,71]. Their effects on growth performance, nutrient utilisation and meat quality depend on insect species, processing method, dietary inclusion level and overall diet formulation.

Microalgae, including *Arthrospira platensis* (spirulina) and *Chlorella vulgaris*, have also been investigated as alternative feed ingredients. In rabbits, supplementation influenced growth, nutrient utilisation and antioxidant-related responses, while reproductive responses varied with supplementation level [72,73]. *Spirulina* supplementation also increased vitamin B12 content in rabbit meat [74]. In dairy cattle, processed *Prototheca moriformis* microalgae meals, partially replacing ground maize, maintained milk yield and composition [75]. When *Spirulina* and *Chlorella vulgaris* were evaluated as supplementary protein sources, milk production was maintained, although feed acceptance and nitrogen-utilisation responses differed from those obtained with rapeseed meal [76]. In dairy cattle, supplementation with 200 g/day of spirulina during early lactation increased milk yield and body-condition scores compared with the control group [77]. In dairy goats, *Chlorella vulgaris* did not significantly affect milk yield, composition or fatty acid profile, although selected antioxidant-related responses differed between treatments [78].

The reviewed evidence indicates that the suitability of alternative feed resources differs among livestock species and production systems. In dairy cattle, agro-industrial by-products can partially replace conventional feed ingredients while maintaining milk production or modifying selected milk-quality characteristics, although responses depend on the ingredient and inclusion level [43,44,45,46,47,48,49,50]. In pigs and poultry, oilseed cakes provide alternative sources of protein and energy, but differences in digestibility, processing characteristics and anti-nutritional factors may limit their inclusion [51,52,53,54,55,56,57,58,59,60,61,62]. The reduction in growth performance or egg production reported with higher camelina cake inclusion in quails and rabbits further illustrates that maintaining product quality does not necessarily indicate nutritional adequacy or acceptable productive performance [63,64,65,66]. Insect meals and microalgae offer additional options for diversifying feed supplies, although their effects on animal performance, nutrient utilisation and product quality vary with the source, processing method, inclusion level and livestock species [67,68,69,70,71,72,73,74,75,76,77,78]. These differences indicate that inclusion levels and productive responses observed in one species or production system should not be directly extrapolated to another.

The nutritional benefits of alternative feed resources must also be considered alongside their processing requirements, safety, cost and environmental implications. Biological processing may improve the feeding value of certain low-quality residues, but its practical suitability depends on substrate characteristics, processing conditions and the consistency of the resulting feed [40,81,82]. Similarly, the use of locally available or recycled biomass does not necessarily reduce the environmental burden of livestock production. Additional processing, transport or changes in animal performance may offset the benefits of replacing conventional ingredients, while some residual materials may have alternative uses in composting, anaerobic digestion or soil amendment [19,38]. The selection of feeding strategies should therefore consider nutrient availability, feed availability, animal requirement, animal performance, feed safety, economic feasibility and environmental impacts under the intended production conditions. These outcomes should be assessed independently where appropriate, rather than assuming that improvements in one indicate corresponding benefits across the proposed One Health pathway.

### 3.3. Feed Safety and Integrated Pathways to Resilient One Health Systems

Feed safety is a prerequisite for sustainable livestock production and public health. Feed-borne hazards, including mycotoxins, pesticide residues, heavy metals and pathogenic microorganisms, can reduce animal health and productivity and may enter the human food chain through meat, milk and eggs [85,86]. However, the presence of a chemical hazard does not necessarily indicate an unacceptable health risk. Risk depends on the toxicological properties of the substance, its concentration and mode of action, the magnitude and duration of exposure, and the tolerance level and susceptibility of the exposed animal or human population [87]. Accordingly, detection of a contaminant should be distinguished from an assessment of its potential adverse health effects. Feed safety should therefore be considered the first stage of food safety. This requires a whole-chain approach incorporating hygienic handling, moisture control, appropriate storage, routine testing, traceability and risk-based quality assurance, particularly when using variable local ingredients, by-products and fermented materials [42,88]. A feed resource cannot be considered sustainable unless its safety, consistency and traceability are demonstrated. Improved forages, legumes, silvopastoral systems, alternative feed resources, precision nutrition and feed-safety governance should be implemented as complementary rather than isolated interventions [6,31,39]. Table 2 summarises the principal feed-resource strategies, their potential benefits and the conditions affecting their implementation.

## 4. Healthy Animals: Nutrition, Animal Welfare, Disease Prevention and Antimicrobial Stewardship

Animal health is an important outcome of nutritional management and also influences the efficiency with which dietary nutrients are utilised. Inadequate nutrient supply, poor feed quality and inappropriate feeding practices may impair animal health and productivity, while disease and environmental stress can alter nutrient requirements and responses to dietary interventions [7,14]. Nutritional management should therefore be considered alongside veterinary care, biosecurity and animal welfare when evaluating animal-health outcomes within the proposed One Health pathway.

### 4.1. Nutrition: The First Line of Defence in Animal Health

Nutritional adequacy supports immune function, metabolic stability, reproduction and gastrointestinal integrity [7,89]. However, animal-health responses to nutritional interventions depend on the initial nutritional status, species, physiological stage, dietary composition and prevailing management conditions. Correcting a nutrient deficiency may restore physiological function, whereas supplementation beyond established requirements does not necessarily provide additional health benefits [89,90,91]. Nutritional interventions should therefore be evaluated according to the specific physiological response or health status outcome they are intended to address.

Diet and feeding systems may also influence welfare-related behaviours, although the mechanisms linking nutritional interventions, gut microbiota and behaviour are not fully established. In pigs, dietary factors and gut microbial composition have been associated with tail-biting behaviour [92], while associations between gut microbiota and feather pecking have been reported in chickens [93]. These findings suggest possible relationships between nutrition, intestinal microbial communities and behaviour, but do not establish that modifying the microbiota through dietary intervention will prevent these behaviours. Nutritional imbalance can also predispose animals to production diseases. In dairy cattle, inadequate transition feeding and mineral imbalances are associated with metabolic disorders such as hypocalcaemia and ketosis, whereas insufficient physically effective fibre and excessive intake of rapidly fermentable carbohydrates increase the risk of subacute ruminal acidosis [94,95]. These conditions are associated with ruminant digestive and metabolic physiology and should not be extrapolated to pigs or poultry. In monogastric animals, dietary fibre influences gastrointestinal function through its effects on digesta passage, microbial fermentation and nutrient utilisation, with responses depending on the fibre source, physicochemical properties and inclusion level [96,97]. Undernutrition during periods of seasonal feed scarcity can also reduce body condition, delay puberty, lower conception rates and impair immune function [7,98]. Nutritional management should therefore account for species-specific requirements, physiological stage and production conditions when evaluating its contribution to animal health.

The gastrointestinal tract represents an important interface between nutrition and health. In ruminants, physically effective fibre stimulates rumination and saliva secretion, contributing to rumen buffering and maintaining conditions for microbial fermentation [95]. In pigs, dietary fibre undergoes microbial fermentation predominantly in the hindgut, particularly the caecum and colon. The resulting short-chain fatty acids can influence intestinal barrier function, microbial composition and local immune responses [96]. In poultry, dietary fibre influences gizzard development, digesta passage and intestinal microbial activity, with microbial fermentation occurring mainly in the caeca. Its effects on nutrient utilisation and intestinal health depend on fibre solubility, particle size and dietary inclusion level [97]. These physiological differences should be considered when interpreting the effects of nutritional interventions on gastrointestinal health and immune function. Probiotics, prebiotics, organic acids, phytochemicals and selected bioactive compounds have been investigated for their effects on gut health and enteric pathogen control [99,100]. However, their efficacy varies with species, dose, production stage, farm hygiene and overall diet quality [101]. For example, Canibe et al. [102] reviewed the nutritional and management approaches for preventing post-weaning diarrhoea in pigs while reducing reliance on antibiotics and the pharmacological levels of zinc oxide. The interventions examined included probiotics, prebiotics, synbiotics, postbiotics, plant-derived compounds, macroalgae, dietary fibre, antimicrobial peptides, specific amino acids, dietary fatty acids and bacteriophages. The authors concluded that combinations of measures are likely to be more effective than reliance on a single intervention [102]. These findings support the consideration of nutritional strategies within preventive animal-health programmes, but their effects on disease incidence and antimicrobial requirements should be evaluated under the specific conditions in which they are applied.

### 4.2. Herd-Health Management, Veterinary Care and Surveillance

Nutritional interventions should be considered alongside preventive veterinary care and herd-health management. Disease, parasitism and metabolic disorders may alter nutrient intake, utilisation and requirements, making it difficult to distinguish the effects of dietary interventions from those of underlying health conditions [13,15]. Regular monitoring of body condition, feed intake, productive performance and disease occurrence can help identify nutritional and health problems. Veterinary diagnosis and treatment remain necessary when disease occurs, while adequate nutrition, hygiene and biosecurity can contribute to reducing preventable disease pressure and the need for antimicrobial treatment [102,103,104].

Monitoring technologies can support the assessment of nutritional and animal-health responses by recording changes in feed intake, rumination, body weight, milk yield and activity [15,32]. Their usefulness depends on data quality, infrastructure and the ability to translate measurements into appropriate feeding or veterinary decisions. In smallholder systems, routine body-condition scoring, feed records, farmer training and access to veterinary services may be more practical than advanced monitoring technologies [105].

### 4.3. Vaccination, Biosecurity and Pathogen Control

Vaccination and biosecurity are central components of preventive animal health. Vaccines can reduce the incidence and severity of infectious diseases, improve herd immunity and decrease the need for antimicrobial treatment when properly selected and administered [101,106]. These measures complement nutritional management by reducing disease pressure and supporting animal health, although adequate nutrition cannot replace disease-specific preventive measures or clinically indicated treatment. Vaccination programmes should be adapted to species, age, physiological status, disease risk, vaccine availability and national regulations, with appropriate veterinary guidance [101,106]. Some diseases are vaccines preventable (Supplementary Table S1). However, their utility is not universal but rather specific to the disease control policy of the country or region.

Biosecurity reduces the probability that pathogens will enter, circulate within or leave a farm. Measures such as controlled animal movement, quarantine, hygienic housing, safe feed and water, and appropriate manure and waste management can reduce pathogen exposure and support animal health [13]. Their implementation depends on the production conditions, available resources and access to veterinary services, particularly in smallholder systems [105]. Wider land-use changes and increased contact among wildlife, livestock and humans may also influence pathogen exposure [107]. These factors are relevant to nutritional interventions because poor feed quality, contamination and underlying disease can modify animal responses to feeding strategies. Preventive measures, including safe feeding practices, vaccination, biosecurity and veterinary oversight, also complement hygienic processing and handling when reducing foodborne risks [3,13,108]. Nutritional interventions should therefore be interpreted alongside these preventive measures rather than as independent determinants of animal health or food safety.

### 4.4. Welfare, Housing and Low-Stress Handling

Animal welfare and nutritional management are interrelated because housing conditions, access to feed and water, stocking density and environmental stress can influence nutrient intake, metabolic requirements and productive performance [14,15,109]. Thermal stress is particularly relevant because it can reduce feed intake and modify physiological responses to dietary interventions. In broilers, heat stress has been associated with reduced growth performance, impaired immune function and changes in meat quality [17,110,111,112], while pigs and rabbits are also susceptible to high ambient temperatures, with adverse effects on feed intake, productive performance and physiological function [113,114,115,116,117]. Nutritional interventions should therefore be interpreted in relation to environmental conditions and welfare status, particularly under thermal stress.

Other management factors can also influence outcomes attributed to feeding strategies. In broilers, environmental enrichment has affected activity, productive performance, breast muscle characteristics and locomotor responses [118,119,120]. Pre-slaughter stress can alter muscle glycogen reserves and post-mortem pH, with consequences for meat quality [16,121]. These factors should be considered when interpreting dietary effects on animal welfare and product quality, as the observed responses may reflect both nutritional and non-nutritional influences.

### 4.5. Breeding and Genetics for Disease Resistance and Resilience

Genetic characteristics can influence animal responses to nutritional and environmental conditions. Selection for disease resistance, resilience, heat tolerance and productive efficiency may improve adaptation to challenging production environments, but these benefits depend on appropriate nutrition and management [111,122,123,124,125]. Conversely, selection for high productive performance may be accompanied by undesirable health or product-quality outcomes; for example, rapid growth and increased breast muscle yield in broilers have been associated with breast muscle myopathies such as white striping, wooden breast and spaghetti meat [126,127,128]. Feeding strategies should therefore be matched to the genetic characteristics and productive potential of animals, rather than assuming that a nutritional intervention will produce comparable responses across breeds or genotypes [122,126].

### 4.6. Antimicrobial Resistance and Responsible Antimicrobial Stewardship

Antimicrobial resistance is a major concern within the One Health framework because resistant microorganisms and resistance genes can circulate among humans, animals and the environment. Antimicrobial use can select resistant microorganisms, while their persistence and transmission are also influenced by infection prevention, hygiene, environmental contamination and other management factors [129,130,131]. The global human-health burden is substantial. The GBD 2021 analysis estimated that bacterial antimicrobial resistance was directly attributable to 1.14 million deaths and associated with 4.71 million deaths worldwide in 2021 [132]. More recent WHO surveillance estimated that approximately one in six laboratory-confirmed bacterial infections caused by selected common pathogens in humans was resistant to antibiotic treatment in 2023 [129]. Between 2018 and 2023, resistance increased in more than 40% of the pathogen–antibiotic combinations monitored [129]. These estimates describe the human-health burden and surveillance trends in the populations and pathogen–antibiotic combinations evaluated; they do not quantify the contribution of livestock production or nutritional interventions to antimicrobial resistance.

Responsible antimicrobial stewardship in livestock should be based on disease prevention, diagnosis and prudent antimicrobial use. Antimicrobials should be administered when clinically indicated, under veterinary guidance, at an appropriate dose and duration, with attention to withdrawal periods, record keeping and susceptibility testing where feasible [104]. The use of medically important antimicrobials for growth promotion is inconsistent with One Health stewardship principles, while routine prophylactic use should be minimised through appropriate nutrition, vaccination, biosecurity, hygiene and animal-welfare management [104,129]. These preventive measures may reduce disease occurrence and the need for antimicrobial treatment. However, a reduction in antimicrobial use does not, by itself, demonstrate a reduction in antimicrobial resistance. Although restrictions on antimicrobial use have been associated with lower resistance prevalence in several animal-production settings, the magnitude and consistency of these responses vary with the antimicrobial class, bacterial species, existing resistance patterns and transmission conditions. Antimicrobial use and resistance should therefore be evaluated as separate outcomes through appropriate treatment records and microbiological surveillance [104,108,129].

Non-antibiotic preventive measures should be viewed as approaches for reducing disease risk and the need for antimicrobial treatment, rather than as direct substitutes for clinically indicated therapy. Vaccines can reduce disease occurrence and consequently antimicrobial use when implemented alongside good management and adequate coverage [101,106]. Fat-soluble vitamins A, D, E and K, water-soluble B-group vitamins, and vitamin C may influence gastrointestinal functionality, particularly during periods of increased susceptibility to gut disorders early in life [12]. A range of natural and biological approaches is also being investigated for their potential to improve gut health and reduce pathogen pressure, including probiotics, prebiotics, organic acids, enzymes, phytochemicals, competitive exclusion products, bacteriophages and antimicrobial peptides, although their effects remain context-dependent [101,106]. Such alternatives should be evaluated for efficacy, safety, consistency, regulatory status and cost-effectiveness before large-scale recommendation. The most reliable strategy is still prevention-led health management, that reduces the need for antimicrobials in the first place.

### 4.7. Extension, Farmer Capacity and One Health Implementation

The adoption of improved feeding strategies depends on farmers’ access to quality feed resources, technical guidance, veterinary services and financial support. In smallholder systems, limited access to feed testing, storage facilities and appropriate processing technologies may constrain the use of alternative feed ingredients and nutritionally balanced diets [13,105]. Extension services and farmer training can support the adoption of feeding practices suited to local feed resources, animal requirements and production conditions [105].

Implementing nutritional interventions within a One Health framework also requires coordination among farmers, animal nutritionists, veterinarians, feed suppliers and food-chain actors. Such coordination is particularly relevant when introducing alternative feed resources, where nutritional composition, contamination risks, feed-processing requirements and potential effects on animal-derived foods need to be assessed together [3,4,108]. The practical value of these interventions ultimately depends on their nutritional suitability, safety, affordability and acceptance under local production conditions.

## 5. Safe and Nutritious Animal-Source Foods: Linking Animal Health with Food Quality, Safety and Public Health

**Figure 3:**
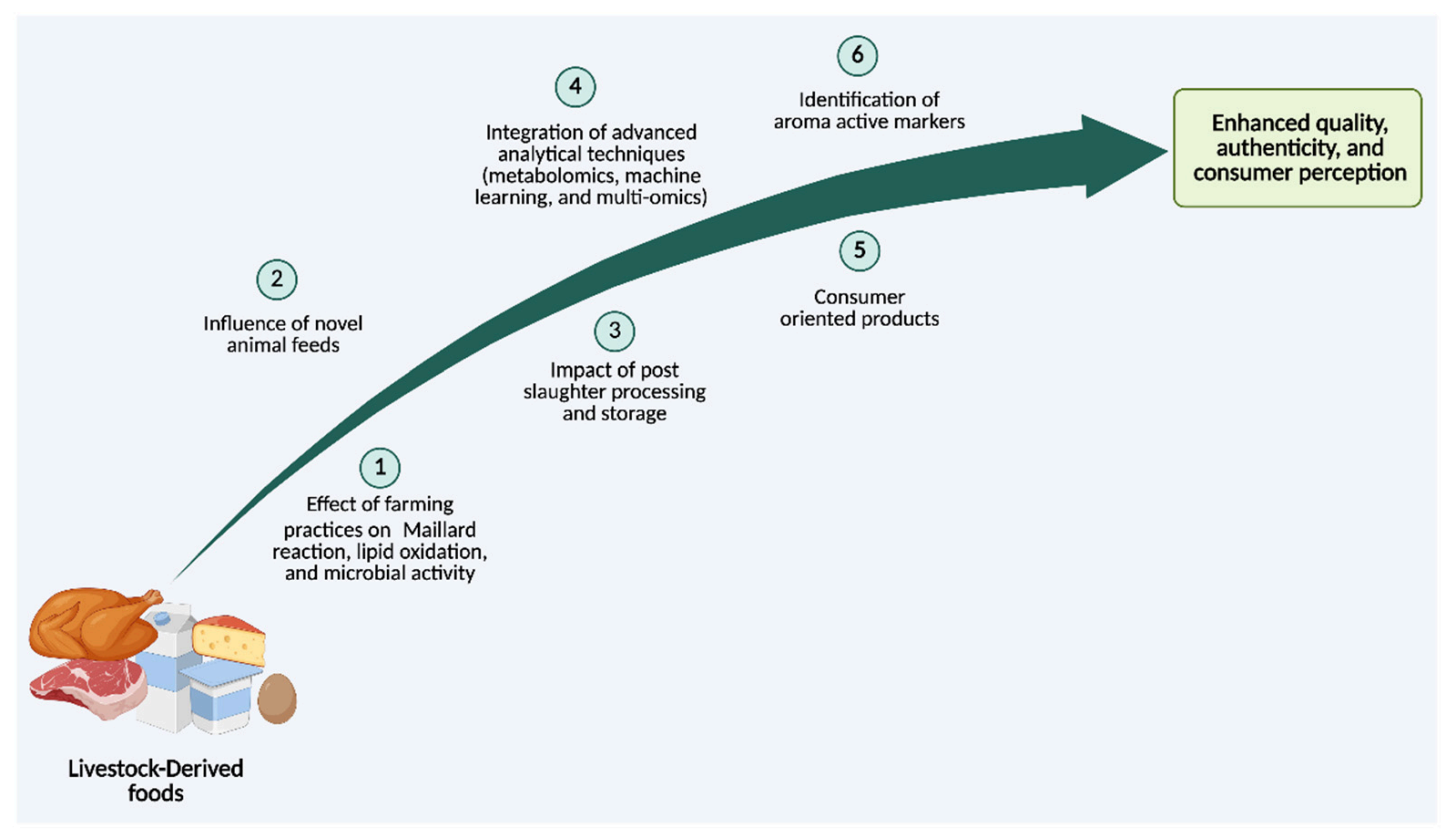
Farm-to-consumption pathway linking animal health, hygienic production, processing, cold-chain management and consumer handling in preserving the nutritional quality and safety of animal-source foods. Modified from Ponnampalam et al. [10].

Safe and nutritious food is the next outcome in the feed-to-food pathway. Better feeds and healthy animals create the biological foundation, but the final value of animal-source foods also depends on hygienic production, disease control, residue prevention, processing, cold-chain management and consumer-level handling. Animal-source foods such as meat, milk, eggs, fish and offal provide high-quality protein and several micronutrients that can be difficult to obtain in adequate amounts from monotonous plant-based diets, particularly vitamin B12, highly bioavailable iron and zinc, calcium, choline, essential amino acids and long-chain omega-3 fatty acids [133,134]. However, their public-health value is realised only when they are produced, processed and consumed safely. The objective is therefore not simply to increase production, but to preserve nutritional quality, microbiological and chemical safety, and consumer acceptability throughout the production-to-consumption chain, as illustrated in Figure 3.

### 5.1. Nutritional Contribution of Animal-Source Foods

Animal-source foods have a distinct role in human nutrition because they combine high-quality protein with a dense supply of bioavailable micronutrients [133]. Meat provides essential amino acids, haem iron, zinc and vitamin B12; milk contributes protein, calcium, phosphorus, riboflavin and bioactive peptides; eggs provide high-quality protein, choline, fat-soluble vitamins and carotenoids; and fish contributes protein, iodine, selenium and long-chain omega-3 fatty acids, depending on species and production system [133,134]. These foods are particularly important for children, pregnant and lactating women, adolescent girls, older adults, and populations with poor dietary diversity, where micronutrient deficiencies and protein inadequacy remain important nutritional concerns [133,134].

Importantly, the nutritional value of animal-source foods is not fixed but can be influenced by animal feeding strategies. Feeding strategies can modify selected nutritional characteristics of animal-derived foods. Dietary inclusion of oilseed-derived ingredients has altered the fatty acid composition of meat, milk and eggs [43,58,60,62], while spirulina supplementation increased vitamin B12 content in rabbit meat [74]. However, these responses do not necessarily occur alongside improvements in productive performance, and their effects depend on the feed ingredient and inclusion level [59,63,64,66].

Recent evidence in goats further demonstrates this feed-to-food linkage, where the complete replacement of maize grain with a self-fermented agro-waste mix improved meat tenderness and oxidative stability, increased n-3 polyunsaturated fatty acid content and produced a more favourable n-6/n-3 fatty acid ratio [135]. These findings demonstrate that dietary interventions can modify selected nutritional and quality characteristics of animal-derived foods, although their wider environmental and economic implications require separate assessment.

The contribution of animal-source foods should nevertheless be interpreted in a context-specific manner. In undernourished populations, improved access to milk, eggs, meat or fish can enhance dietary diversity and nutrient adequacy [133]. In populations with excessive intake of processed meat or high total energy intake, moderation and dietary balance become more important [133]. Animal-source foods should therefore not be characterised as universally beneficial or universally harmful. Their health value depends on the type of food, degree of processing, amount consumed, background diet, nutritional status of the population and sustainability of the production system [133].

### 5.2. Microbiological Safety of Animal-Derived Foods

Animal-derived foods are highly nutritious, but this nutrient richness can also support microbial growth when hygiene and temperature control are inadequate. Important foodborne hazards associated with animal-source foods include *Salmonella*, *Campylobacter*, pathogenic *Escherichia coli*, *Listeria monocytogenes*, *Brucella*, *Staphylococcus aureus* and several parasites, depending on the species, production system and handling practices [13,136]. Raw or undercooked poultry, raw milk, inadequately cooked meat, contaminated eggs and ready-to-eat products exposed to cross-contamination are important routes through which foodborne pathogens may reach consumers [136].

Microbiological safety must therefore be controlled throughout the food chain. At the farm level, animal-health monitoring, biosecurity, hygienic milking, clean water, safe feed, manure management and separation of sick animals can reduce pathogen entry into the food chain [3,13]. During slaughter and processing, hygienic dressing, prevention of faecal contamination, equipment sanitation, worker hygiene, use of potable water and separation of raw and cooked products are essential control measures [137]. At retail and household levels, refrigeration, prevention of cross-contamination, adequate cooking and safe storage are equally important because foodborne risks may also arise after products leave the farm or processing plant [136].

Milk safety deserves particular attention because milk is widely consumed by children and nutritionally vulnerable groups. Hygienic milking, udder-health control, clean containers, rapid cooling and pasteurisation are important for reducing milk-borne pathogens [136,137]. Similarly, meat safety depends on healthy animals presented for slaughter, ante-mortem and post-mortem inspection, hygienic carcass dressing, chilling and prevention of cross-contamination [137]. Egg safety requires flock health, clean nesting and collection practices, shell integrity, hygienic storage and appropriate cooking, particularly where *Salmonella* risk is present [136].

### 5.3. Chemical Hazards, Residues and Feed-to-Food Carry-Over

Food safety also requires control of chemical hazards. Foods of animal origin may contain contaminants originating from feed, water, the environment, veterinary drug use or processing. Feed-related chemical hazards include mycotoxins, pesticide residues, heavy metals and persistent environmental contaminants [8,86]. However, their transfer into animal-derived foods is not uniform and depends on the chemical properties of the substance, the amount and duration of exposure, absorption, metabolism, excretion, animal species and physiological stage. The distribution of contaminants also differs among edible products and tissues. For example, aflatoxin B1 in contaminated feed can be metabolised to aflatoxin M1 and excreted in milk, with transfer influenced by dietary aflatoxin intake, milk yield and individual animal characteristics [138]. In contrast, persistent lipophilic contaminants, such as dioxins and dioxin-like polychlorinated biphenyls, can accumulate in body fat and liver and may be transferred into milk and eggs [139]. These differences indicate that the detection of a contaminant in feed does not establish its concentration in edible products or the resulting consumer exposure. Health risk depends on the toxicological properties of the substance, its concentration in the food consumed, the amount and duration of dietary exposure, and the susceptibility of the exposed population [87]. Feed monitoring, appropriate storage, contamination control and ingredient traceability are therefore necessary to limit exposure before contaminants enter the food chain.

Veterinary drug residues are another concern in animal-source foods. Antimicrobials, anthelmintics, anti-inflammatory drugs and other veterinary medicines are necessary for animal health when appropriately used, but unacceptable residues may occur following improper administration or failure to observe withdrawal periods [104,140]. Residue concentrations depend on the drug and its metabolites, administered dose, route and frequency of administration, species-specific metabolism, tissue distribution and time elapsed since treatment. Consequently, residue depletion may differ among muscle, liver, kidney, fat, milk and eggs [141]. Processing can also modify residue concentrations through chemical degradation, redistribution among product fractions or changes in moisture content. However, these effects vary with the compound, food matrix and processing conditions, and processing cannot be relied upon to eliminate residues or compensate for inadequate control before harvest [141]. Maximum residue limits (MRLs) for specified pesticide and veterinary drug residues, as well as maximum levels (MLs) for contaminants such as mycotoxins and certain heavy metals, provide regulatory benchmarks for controlling chemical contamination in food and feed [85,142]. Applicable limits are substance- and commodity-specific and should be considered alongside exposure assessment and the relevant national regulations. Exceedance of an applicable limit indicates regulatory non-compliance but does not, by itself, establish that an adverse health effect has occurred. Conversely, compliance does not imply the complete absence of risk under all exposure conditions. Residue prevention requires veterinary oversight, appropriate drug administration, treatment records, observance of withdrawal periods and residue monitoring [104,140]. Veterinary drug residues and antimicrobial resistance represent distinct food-safety concerns and should be assessed separately.

Chemical deterioration may also occur during processing and storage. Lipid oxidation can reduce sensory and nutritional quality and generate undesirable oxidation products in meat and other lipid-containing animal foods, particularly when the content of unsaturated fatty acids is increased without adequate antioxidant protection and packaging [10]. Inappropriate smoking, heating or frying may also generate processing-related contaminants, while poor packaging and storage can accelerate rancidity, discolouration and deterioration. Nutritional quality and safety must therefore be preserved together; a food cannot be regarded as high quality if it is nutritionally rich but microbiologically unsafe, chemically contaminated or unstable during storage.

### 5.4. Processing, Preservation and Cold-Chain Management

Post-harvest processing determines whether the nutritional and safety advantages created on farms are retained until consumption. Good hygienic practices and hazard analysis and critical control point-based systems provide structured approaches for identifying, preventing and controlling hazards during food processing and handling [137]. For animal-source foods, important interventions include milk pasteurisation, hygienic slaughter, rapid carcass chilling, validated cooking, fermentation where appropriate, drying, salting, packaging, refrigeration and frozen storage. When correctly applied, these interventions reduce microbial load, slow spoilage and extend shelf life [136,137].

Cold-chain management is particularly important because meat, milk, eggs and fish are highly perishable. Temperature abuse during transport, retail display or household storage can permit the rapid growth of spoilage and pathogenic microorganisms [136]. Maintaining an uninterrupted cold chain reduces microbial proliferation, preserves sensory quality and limits economic losses due to spoilage. However, cold-chain infrastructure may be weak in smallholder, informal and remote market systems. In such settings, practical interventions such as rapid local collection, clean containers, shaded transport, evaporative cooling, community chilling facilities, hygienic processing and consumer education may be more feasible than relying on high-cost technologies alone.

Processing should also preserve nutritional quality. Excessive heating and inappropriate processing conditions can reduce heat-sensitive nutrients, while lipid oxidation during storage can adversely affect flavour, pigments and overall product quality [10]. Conversely, properly controlled processing can improve the safety and digestibility of animal-source foods and reduce pathogen exposure. Pasteurisation, for example, improves milk safety, while controlled fermentation can enhance product stability and, in some cases, functional characteristics [137]. Processing should therefore be designed not merely to produce marketable products but to preserve their nutritional, sensory and safety attributes.

### 5.5. Quality Assurance, Traceability and Consumer Confidence

Ensuring food quality and safety requires integrated systems rather than isolated interventions. Farm records, animal-health status, feed safety, veterinary drug use, compliance with withdrawal periods, hygienic processing, residue monitoring, microbial testing, cold-chain verification and consumer information should form part of quality-assurance programmes [42,137]. Traceability is particularly important when feed contamination, disease outbreaks, veterinary drug residues or foodborne illness require rapid identification of the source [137]. Without effective traceability, unsafe products may remain in circulation and consumer confidence can be undermined.

Digital tools can strengthen traceability but cannot replace fundamental hygiene, regulatory oversight and quality-control practices. Blockchain, sensors, electronic records and data platforms may improve transparency in organised value chains, but their effectiveness depends on data quality, infrastructure, regulatory acceptance and participation by farmers and other value-chain actors [143]. In many low-resource systems, strengthening basic controls such as hygienic production, veterinary oversight, observance of withdrawal periods, cold-chain access, laboratory capacity and market-level inspection remains a priority [13,136]. Quality-assurance systems should therefore be appropriate to the production environment while maintaining consumer protection as their primary objective.

Consumer confidence depends on demonstrable safety, consistent quality and credible regulation. Animal-source foods are increasingly expected to be safe, nutritious, ethically produced and environmentally responsible [5,133]. Meeting these expectations requires clear communication and evidence-based standards rather than unsupported claims. Terms such as “natural”, “antibiotic-free”, “organic”, “welfare-friendly” and “sustainable” should therefore be supported by the appropriate production standards, verification and, where relevant, testing.

Overall, the safe and nutritious production of animal-source foods requires control of both pre-harvest and post-harvest conditions. Meat, milk, eggs and other animal-derived foods can provide genuine nutritional benefits only when they originate from healthy animals receiving safe feeds and are accompanied by hygienic production, residue control, appropriate processing, cold-chain management and quality assurance. Within the One Health pathway, animal-source foods provide a direct link between animal health and human health. The next stage is therefore to consider how livestock production and value chains influence livelihoods, community resilience and environmental health.

## 6. Livelihoods, Circularity and Sustainable One Health Outcomes in Healthy Communities and a Healthy Planet

Improved feeding strategies can influence livestock productivity, animal health, food availability and farm income, with potential implications for rural livelihoods and environmental sustainability. Livestock systems also influence household income, employment, gender roles, rural resilience, nutrient cycling, landscape management and climate adaptation [144,145]. Thus, the outcome of the livestock feed-to-food pathway is not simply more food, but better food supported by economically viable, socially inclusive and environmentally responsible production systems. From a One Health perspective, this requires recognition of the livelihood importance of livestock while reducing environmental burdens, zoonotic and foodborne risks, and pressure on natural resources [3,146].

### 6.1. Livestock, Livelihoods and Rural Economic Resilience

Livestock play multiple roles in rural livelihoods by providing food, income, manure, draught power, savings, insurance, transport, cultural value and social security, particularly in smallholder and mixed crop–livestock systems [145,147]. Livestock support the livelihoods of approximately 1.3 billion people globally and contribute substantially to agricultural production in both developing and developed economies [144]. For poor and vulnerable households, animals can function as living assets that help smooth consumption, support expenditure on education and healthcare, buffer crop failure, illness or market shocks, and provide emergency income when sold [148]. This role is particularly important where formal financial institutions, insurance and stable markets are limited. Goats, sheep, poultry, pigs, cattle and buffaloes provide flexible assets that households can accumulate, reproduce and liquidate according to need [145,148]. Rabbits can also contribute to household resilience because their high reproductive efficiency, rapid growth, limited space requirements and comparatively low production costs enable the production of high-quality animal protein and income with modest investment [149,150]. Their capacity to utilise forages and locally available feed resources further supports their suitability for resource-constrained production systems. Access to affordable and nutritionally adequate feed also influences livestock productivity, production costs and the economic contribution of animals to rural households.

Livestock-based resilience should, however, not be assumed. Disease outbreaks, feed shortages, drought, market disruption, theft, price volatility and limited access to veterinary services can rapidly turn livestock from an asset into a liability [1,148]. The livelihood value of livestock therefore depends on reliable animal-health services, feed security, market access, risk-management mechanisms and equitable participation in value chains. Livestock interventions can improve household welfare, but their effects are context-specific. Evidence from livestock-transfer programmes, graduation programmes and livestock insurance indicates that livestock can support income generation, asset accumulation, food security and women’s empowerment when interventions are accompanied by training, veterinary support, market access and financial services [148]. Simply providing animals without ensuring feed availability, disease control, market access and household decision-making capacity may result in limited or unsustainable benefits. The contribution of nutritional interventions to rural livelihoods should therefore be evaluated in relation to feed availability, animal performance, production costs and household income under local production conditions.

### 6.2. Employment, Value Chains and Inclusive Economic Opportunities

Livestock value chains generate employment well beyond primary production. Feed production, forage cultivation, veterinary services, artificial insemination, transport, animal marketing, slaughter, milk collection, meat and dairy processing, cold-chain logistics, retailing and by-product utilisation all create income opportunities in rural and peri-urban economies [151,152]. These activities are particularly important where crop income is seasonal and alternative employment is limited. The use of locally available feed resources and agro-industrial by-products may also create opportunities for feed production and processing, depending on raw material availability, processing costs and market demand. Sustainable livestock value chains can therefore contribute to rural transformation by converting locally available biological resources into food products, services, employment and income.

Small-scale livestock producers often face disadvantages within value chains because of weak bargaining power, inadequate infrastructure, limited access to credit and veterinary services, lack of cold-chain facilities and dependence on informal traders [151]. These constraints can reduce farm-gate returns and limit producers’ ability to benefit from increasing demand for animal-source foods. Sustainable value-chain development therefore requires producer organisation, transparent pricing, quality-based payment systems, reliable input supply, animal-health services, processing infrastructure, market information and inclusive governance [151]. Without such support, growth in livestock markets may disproportionately benefit intermediaries rather than primary producers.

Women and young people are central to inclusive livestock development. Women are often extensively involved in small-ruminant, poultry, dairy and backyard livestock systems, yet their control over income, land, credit, training and decision-making may be constrained by social and institutional barriers [153,154]. Livestock interventions can contribute to women’s empowerment when they strengthen ownership rights, access to services, participation in decision-making, control over income and access to labour-saving technologies [148,154]. Similarly, meaningful engagement of young people in livestock value chains requires skills development, entrepreneurship support, digital literacy, access to finance, market linkages and dignified working conditions. Access to feed resources, training and markets may influence the extent to which nutritional interventions contribute to household income and community well-being.

### 6.3. Nutrition-Sensitive Livestock Systems and Community Well-Being

Safe animal-source foods can contribute to nutrition security, but community-level benefits depend on accessibility, affordability, cultural acceptability and intra-household distribution. Livestock-owning households may not necessarily consume more animal-source foods when products are primarily sold for income, reserved for particular occasions, controlled by specific household members or priced beyond local purchasing power [133,148]. Nutrition-sensitive livestock development should therefore connect production with dietary education, women’s participation in household decision-making, food safety, market access and affordability.

Livestock can influence community well-being through both direct and indirect pathways. Direct pathways include household consumption of milk, eggs, meat or offal, whereas indirect pathways operate through livestock-derived income used to purchase diverse foods, healthcare, education and other household needs [148]. These benefits are more likely to be realised when animal health, productivity and market access are stable. They may be weakened by disease outbreaks, feed shortages, low product prices or food-safety concerns. Nutrition-sensitive livestock systems should therefore integrate production, safety, income and behaviour-change components rather than assuming that increasing livestock numbers will automatically improve household diets.

Community well-being also depends on limiting health risks associated with livestock production. Poorly managed systems may increase exposure to zoonotic pathogens, antimicrobial-resistant bacteria, manure-borne contaminants and environmental pollution [3,13]. These risks do not negate the contribution of livestock but emphasise the importance of appropriate husbandry, safe manure handling, veterinary oversight, clean water, food hygiene and public-health coordination. A One Health approach is particularly relevant because it recognises nutrition, livelihoods, animal health, environmental sanitation and public health as interconnected community outcomes rather than isolated sectors [155,156].

### 6.4. Circular Livestock Systems, Manure Management and Resource Efficiency

Livestock can contribute to circular agrifood systems by converting human-inedible biomass into food and returning nutrients to crop production [157,158]. This role is particularly relevant when animals utilise grasses, crop residues, agro-industrial by-products and other biomass streams unsuitable for direct human consumption, as discussed in Section 3. Circular livestock systems can reduce waste, improve resource-use efficiency and strengthen connections among crop, feed, animal and soil systems [38,157]. However, the use of residual biomass as feed does not necessarily reduce the overall environmental burden. Life-cycle assessments should consider the environmental impacts of collection, processing, drying, transport and feed utilisation, together with changes in animal performance and manure-related emissions. Comparisons should use consistent system boundaries and functional units and account for alternative uses of the biomass, such as composting, anaerobic digestion or soil amendment [157,158]. The environmental value of circular feeding therefore depends on the production system and the extent to which resource recovery offsets additional inputs and emissions.

Insect farming provides one example of circular bioeconomy by converting selected low-value biomass streams into protein- and lipid-rich ingredients [159]. Insects such as *Tenebrio molitor* and *Hermetia illucens* can bioconvert authorised agro-industrial by-products and former food materials into nutrient-rich biomass, contributing to waste valorisation and resource-use efficiency [160,161]. Insect-derived ingredients can partially substitute conventional protein sources in livestock diets, with potential implications for the nutritional characteristics of animal-derived foods, as discussed in Section 3. Insect production also generates frass that may be recycled as an organic fertiliser, further contributing to nutrient cycling [162,163]. Recent research has additionally explored insect frass as an alternative bedding material for poultry, providing another potential route for valorising insect-production residues [164]. The environmental performance of insect-derived feed ingredients also depends on substrate selection, processing energy, drying requirements and the conventional feed ingredients being replaced; waste valorisation alone does not establish a net environmental benefit [159,160,161].

Manure is central to circular livestock systems because it contains organic matter, nitrogen, phosphorus, potassium and micronutrients that can support soil fertility and crop productivity when appropriately managed [165]. It can also be used for composting, biogas generation and nutrient recovery, contributing to fertiliser substitution and renewable-energy production [157,165]. Conversely, poorly stored or excessively applied manure can contribute to methane and nitrous oxide emissions, ammonia, odour, pathogen dissemination, veterinary drug residues and nutrient runoff [165,166]. Manure should therefore be regarded as a biological resource that requires planned management rather than simply as a waste product or an automatically beneficial resource.

Effective manure management involves appropriate collection, storage and treatment, followed by application at suitable rates, times and distances from water sources [165]. Composting can stabilise organic matter and reduce pathogen loads, while anaerobic digestion can recover methane for energy and produce digestate for nutrient recycling [157,165]. These approaches nevertheless require investment, technical knowledge and maintenance. For smallholders, simpler measures such as compost pits, covered storage, site-specific application and community-level biogas systems may be more practical than complex industrial technologies. Circularity strategies should therefore be adapted to farm scale, climate, labour availability, technical capacity and local markets.

### 6.5. Climate Resilience and Planetary Health

Climate change affects livestock production, while livestock agrifood systems also contribute to greenhouse gas emissions. Heat stress, drought, flooding, pasture degradation and water scarcity can adversely affect animal productivity, reproductive performance and survival [1]. At the same time, livestock systems generate greenhouse gases through enteric fermentation, manure management, feed production, land-use change, energy use and transport [166,167]. This dual relationship makes livestock relevant to both climate adaptation and mitigation, but appropriate responses need to be context-specific, socially feasible and economically realistic.

Climate-mitigation strategies should aim to reduce emissions intensity while supporting productivity, animal welfare and resilience. Improvements in feed quality, herd health, reproductive efficiency, manure management, genetic adaptation, survival and value-chain efficiency may reduce greenhouse gas emissions per unit of animal product [166,167]. However, a reduction in emissions intensity does not necessarily indicate a reduction in absolute emissions. Total emissions may remain unchanged or increase if production expands or if additional emissions associated with feed processing, energy use, transport and manure management offset efficiency gains. The environmental effects of nutritional interventions should therefore be evaluated across relevant life-cycle stages, using consistent functional units and system boundaries, while considering both emissions intensity and absolute emissions where appropriate [166,167]. Climate-smart livestock development also requires broader food-system measures, including reduction of losses, effective land-use governance and ecosystem protection.

Land, water and biodiversity are also central to planetary health. Ecosystem degradation associated with poorly managed grazing, expansion of feed crops, deforestation and nutrient pollution may be reduced in appropriate contexts through well-managed grazing, silvopastoral systems, improved pasture management and integrated crop–livestock systems [20,168]. The outcome depends strongly on management. Livestock may contribute either to landscape degradation or to ecological restoration depending on stocking density, grazing duration, feed sources, manure and water management, and land-use policy. Assessments of livestock and planetary health should therefore be based on production context and evidence rather than generalised assumptions either for or against livestock production.

### 6.6. Governance, Market Access and Equitable Implementation of One Health

Effective governance is central to sustainable livestock transformation. Policies, institutions, services and incentives that support farmers while safeguarding consumers are necessary to sustain better feeds, healthy animals and safe foods [144,146]. Important enabling conditions include veterinary infrastructure, feed and food-safety regulation, antimicrobial stewardship, disease surveillance, extension services, laboratory capacity, quality standards, traceability, animal-welfare guidance and environmental safeguards [3,151]. These services are often least developed in areas where livestock make major contributions to rural livelihoods, creating a persistent gap between policy objectives and implementation at the farm level.

Market access is also an important component of governance. Markets should provide opportunities for smallholders to sell livestock products while protecting public health and environmental quality [151]. Informal markets remain important for many low-income consumers and small-scale producers, but they may have limited access to cold-chain facilities, inspection, traceability and hygienic infrastructure. Formalisation that does not recognise their livelihood functions may exclude vulnerable producers, whereas failure to address food-safety risks may harm consumers. Policies should therefore balance market accessibility and affordability with hygiene, veterinary services, infrastructure, inspection and training [13,151].

One Health implementation requires coordination among farmers, veterinarians, animal nutritionists, public-health professionals, environmental agencies and food-chain actors [3,156]. At farm level, nutritional management and preventive veterinary care can be integrated through feed-quality assessment, animal-health records, treatment monitoring and farmer training. These activities can be evaluated using indicators such as feed intake, productive performance, disease incidence, antimicrobial use and feed-contamination results, depending on the intervention. At the community level, veterinary and public-health services can coordinate disease surveillance, laboratory testing, outbreak reporting and safe manure management, with farmers participating in identifying local priorities and in reporting health and management problems [3,105,156]. Evaluation should consider participation in these programmes, access to veterinary and diagnostic services, disease occurrence and the implementation of recommended preventive measures.

At the value-chain level, feed suppliers, livestock producers, processors and regulatory authorities can coordinate feed testing, traceability, residue monitoring and food-safety controls [42,137,151]. Their implementation can be assessed through compliance with relevant feed and food-safety requirements, contamination and residue-monitoring results, product traceability and documented corrective actions. Resource allocation should reflect locally identified risks and implementation capacity, including the availability of trained personnel, laboratory services, feed-testing facilities, veterinary care and processing infrastructure. In resource-constrained systems, investments should be prioritised according to the risks addressed, expected benefits, costs and feasibility of implementation. Evaluation should distinguish the adoption of recommended practices from demonstrated improvements in animal health, food safety and environmental outcomes, using comparable baseline and follow-up measurements where feasible. Such assessments would help determine whether coordinated interventions produce measurable benefits under different production conditions.

### 6.7. Integrated Pathway from Better Feeds to Healthy Communities and a Healthy Planet

The proposed feed–animal–food–community–environment pathway integrates several relationships through which nutritional interventions may contribute to One Health outcomes. Nutritional adequacy and feed safety can influence animal health and productivity, while dietary composition can modify selected nutritional and quality characteristics of animal-derived foods. Experimental evidence is available for several animal-level responses, including changes in feed intake, nutrient utilisation, productive performance and selected characteristics of meat, milk and eggs following dietary interventions. In contrast, improvements in clinical health, food safety, human nutritional status, community well-being and environmental outcomes cannot be inferred solely from these responses and require assessment using the relevant outcome measures. The translation of animal-level responses into wider One Health benefits also depends on veterinary care, food processing and handling, market access, resource management and production-system characteristics. For example, improvements in the nutritional composition of animal-derived foods may not translate into better human nutrition when the foods are inaccessible or unaffordable. Similarly, improved feed efficiency may reduce resource use per unit of animal product without reducing the absolute environmental burden of the production system. Evidence supporting individual relationships within the proposed pathway is more extensive than evidence demonstrating the complete sequence of downstream outcomes within the same experimental or analytical framework. The pathway should therefore be interpreted as an integrated framework for evaluating nutritional interventions, rather than as a universally established sequence of effects.

## 7. Conclusions and Future Directions: Integrating the Feed–Animal–Food–Community Pathway

Animal nutrition should be viewed as an integrated systems intervention rather than merely as a production input. The contribution of balanced animal nutrition to One Health depends on how feeding strategies affect animal health, food quality and safety, resource use and environmental outcomes under different production conditions. The One Health Joint Plan of Action emphasises collaboration across plant, animal, human and environmental health, and animal nutrition lies naturally at this interface because feed systems connect crops, livestock, food chains, rural economies and the environment [3]. Improved forages, crop residues, alternative protein sources, agro-industrial by-products and precision nutrition can contribute to more efficient feed utilisation, but their suitability depends on nutritional adequacy, feed safety, economic feasibility and local production conditions [1,6,9]. The use of agro-industrial by-products as feed should also be evaluated against alternative uses, such as composting or energy generation, rather than assuming an environmental benefit from their utilisation in livestock diets [5,38,157].

The feed–animal–food relationship remains insufficiently addressed in much of livestock research, where emphasis is often placed primarily on feed efficiency and production outcomes such as growth, milk yield or egg production. Comparatively less information is available on how feeding strategies simultaneously influence animal health, reproduction, welfare, antimicrobial use, food quality and safety, and ultimately human health. These outcomes should be evaluated together where appropriate to determine whether improvements in animal performance are accompanied by benefits in other components of the One Health pathway. However, the strength of evidence supporting individual relationships varies, and improvements in one outcome should not be assumed to produce corresponding benefits throughout the pathway.

The use of alternative feed resources also involves nutritional, safety, economic and environmental trade-offs. Local feed resources, recycled ingredients, crop residues and agro-industrial by-products can support circular livestock systems, but their composition may vary considerably and they may contain undesirable contaminants or toxins [42,88]. Their adoption therefore requires assessment of ingredient quality, nutritional suitability, processing requirements and contamination risks. Similarly, improved feed efficiency may reduce resource use and emissions per unit of animal product without necessarily reducing the absolute environmental burden of the production system. Feeding strategies should therefore be evaluated in relation to animal health, welfare, reproduction, species (animal) performance and environmental outcomes under the conditions in which they are implemented [1,9,126].

Nutritional management should also be considered alongside veterinary care, biosecurity, animal welfare and responsible antimicrobial use. Although adequate nutrition and preventive-health measures may reduce disease occurrence and the need for antimicrobial treatment, these outcomes do not necessarily demonstrate a reduction in antimicrobial resistance [101,104,106,129]. Their contribution to One Health should therefore be assessed through appropriate animal-health and antimicrobial-use indicators, with resistance outcomes evaluated separately where relevant.

Animal-source foods provide high-quality protein and important bioavailable nutrients and can make a valuable contribution to the diets of populations vulnerable to micronutrient deficiencies [133,134]. However, improvements in the nutritional quality of animal-derived foods do not necessarily translate into better human nutrition unless food safety, affordability, accessibility and dietary practices are also considered. Similarly, environmental benefits depend on how feed resources, nutrient losses, manure and other production inputs are managed across the livestock value chain. The proposed feed–animal–food–community–environment pathway therefore provides a framework for assessing these interconnected outcomes, while recognising that evidence supporting the complete sequence of downstream effects remains limited. Future evaluations of nutritional interventions should consider animal performance and health alongside food quality and safety, economic feasibility and environmental impacts, as appropriate to the research question.

## Figures and Tables

**Table 1:** Representative studies on agro-industrial by-products and alternative feed resources in different livestock species.

Feed Resource	Livestock Species	Dietary Inclusion or Treatment	Main Outcomes	Key References
Citrus pulp	Dairy cattle	9% and 18% of dietary DM	Increased milk polyphenol and flavonoid contents and antioxidant capacity. The 18% treatment increased monounsaturated fatty acids and reduced saturated fatty acids in milk fat.	[43]
Dried grape pomace	Dairy cattle	15% of the diet	Milk fat, protein and casein contents were maintained, while lactose and β-lactoglobulin contents increased. Plasma polyphenol content increased, but milk polyphenol content was not significantly affected.	[44]
Dried apple pomace	Dairy cattle	Replacement of 25%, 50% and 75% of maize in the concentrate	Milk yield and most milk-composition traits were maintained, whereas fat-corrected milk yield and milk fat yield decreased with increasing apple pomace inclusion.	[45]
Dried olive pomace	Dairy cattle	10% of dietary DM	Modified the fatty acid composition and aromatic profile of milk and cheese.	[46]
Pressed oilseed cakes (peanut, sunflower, palm kernel, rapeseed and hempseed)	Dairy cattle and other ruminants	Partial replacement of conventional protein ingredients [47,48]; increasing hempseed cake inclusion [50]	Milk production was maintained with peanut, sunflower and palm kernel cakes, while selected milk fatty acid proportions differed among treatments [47]. Rapeseed cake modified milk fatty acid composition [48]. Hempseed cake showed inclusion-dependent effects on milk production, composition and crude protein-use efficiency [50]. The review [49] describes variation in nutrient composition, ruminal metabolism and productive responses among minor oilseed cakes.	[47,48,49,50]
Canola, camelina, rapeseed, linseed and hempseed cakes	Growing pigs	Ingredient-specific nutrient-digestibility and energy-value evaluations	Processing conditions influenced canola cake digestibility [51]. Amino acid digestibility differed among camelina products [52,53] and cold-pressed oilseed cakes [54]. The energy value and additivity of dietary energy contributions were evaluated for camelina cake [55].	[51,52,53,54,55]
Camelina oil cake	Fattening pigs	12% of the diet, replacing sunflower meal	Productive performance was maintained, while selected metabolic, inflammatory and antioxidant-related responses were modified.	[56]
Hydrothermally processed soybean and rapeseed ingredients	Growing-finishing pigs	Comparative feeding trial	Processing and oilseed source influenced selected performance, carcass and backfat fatty acid characteristics.	[57]
Hempseed meal, rapeseed, linseed and hempseed cakes	Laying hens	Hempseed meal at 5%, 10% and 20% [58]; oilseed cakes at 5%, 10% and 15% [59]	Hempseed meal did not significantly affect egg production, feed intake, feed efficiency or egg quality, while increasing selected polyunsaturated fatty acids in eggs [58]. At 15% oilseed cake inclusion, selected laying-performance indices were adversely affected compared with lower inclusion levels [59].	[58,59]
Camelina cake	Broiler chickens	10% of the diet	Growth performance was maintained, while n-3 PUFA deposition in meat increased and the n-6/n-3 ratio decreased.	[60]
Camelina meal with chromium supplementation	Broiler chickens	3% camelina meal with 0, 200 or 400 µg Cr/kg diet as chromium picolinate	Camelina meal increased n-3 PUFA content and reduced the n-6/n-3 ratio in breast meat. Chromium supplementation increased breast meat protein, Zn and Fe content and reduced crude fat and TBARS values. Growth performance was not significantly affected by either dietary intervention.	[61]
Camelina oil and camelina cake	Laying hens	4% camelina oil or 10% camelina cake	Increased n-3 PUFA deposition in egg yolk, while selected laying-performance, egg-quality and sensory responses differed between treatments.	[62]
Camelina cake from different lines	Growing and laying quails	15% of the diet in both studies	Growth performance was adversely affected during the early growing period in growing quails [63]. In laying quails, final body weight and feed intake were not significantly affected, whereas egg production rate and egg weight decreased [64].	[63,64]
Hempseed cake	Adult male rabbits	5% and 10% of the diet	Body-weight gain and selected sperm characteristics were not significantly affected. Haematological and biochemical responses were also evaluated.	[65]
Camelina cake	Growing rabbits	5% and 10% of the diet, replacing rapeseed cake	Growth performance was maintained at 5% inclusion, whereas 10% inclusion reduced feed intake and growth performance.	[66]
Black soldier fly larvae meal (*Hermetia illucens*)	Broiler quails	10% and 15% of the diet, partially replacing soybean meal and soybean oil	Growth performance, carcass yield and most nutrient-digestibility parameters were not significantly affected. Ether extract digestibility decreased at 10% inclusion. Both inclusion levels reduced breast meat ultimate pH, while 15% inclusion increased cooking loss compared with the control.	[67]
Yellow mealworm meal (*Tenebrio molitor*)	Broiler chickens	2%, 4% and 8% dried mealworm meal [68]; 2.5% and 5% larvae meal [69]	Growth responses differed according to inclusion level, with favourable starter-phase responses reported at 4% [68] and 2.5% [69]. Intestinal morphometric characteristics were not significantly affected in the latter study [69].	[68,69]
Black soldier fly larvae meal (*Hermetia illucens*)	Broiler chickens	6% and 12% of the diet [70]; 5% during the starter phase and 10% during the grower phase [71]	Growth performance and selected meat-quality traits were maintained at the tested inclusion levels, although some carcass and nutrient-utilisation responses differed among treatments [70]. Overall growth performance and most meat-quality parameters were not significantly affected, while selected carcass and caecal microbiota responses differed [71].	[70,71]
*Arthrospira platensis* (spirulina) and *Chlorella vulgaris*	Growing rabbits	Both algae at 300 and 500 mg/kg diet	Growth performance and feed conversion improved, particularly with 500 mg/kg *Arthrospira* and 300 or 500 mg/kg *Chlorella*. Glutathione peroxidase activity increased with algal supplementation, while intestinal structure remained normal across treatments.	[72]
*Arthrospira platensis* (spirulina)	Doe rabbits	0.5, 1.0 and 1.5 g/doe/day	Supplementation at 1.0 and 1.5 g/doe/day increased nutrient digestibility, milk yield, litter size and litter weight at weaning compared with the control. Gestation length and individual kit weight at weaning were not significantly affected.	[73]
*Arthrospira platensis* (spirulina), alone or combined with thyme	Growing rabbits	5% spirulina and/or 3% thyme	Spirulina supplementation increased vitamin B12 content in the longissimus muscle. Carcass composition and selected meat-quality characteristics were also evaluated.	[74]
Processed *Prototheca moriformis* microalgae meal, *Arthrospira platensis* and *Chlorella vulgaris*	Dairy cattle	Processed microalgae meal at 91.8 g/kg diet, partially replacing ground maize [75]; microalgae as supplementary protein sources [76]	Processed microalgae meal maintained milk yield and composition and increased ether extract digestibility [75]. Microalgal protein supplementation maintained milk production, but feed acceptance and nitrogen-utilisation responses differed from those obtained with rapeseed meal [76].	[75,76]
*Arthrospira platensis* (spirulina)	Dairy cattle	200 g/cow/day for 90 days during early lactation	Supplemented cows had higher body-condition scores (*p* < 0.01) and greater milk yield (34 versus 28 kg/day; *p* < 0.05) than control cows during early lactation.	[77]
*Chlorella vulgaris*	Dairy goats	10 g/kg concentrate	Milk yield, composition and fatty acid profile were not significantly affected. Selected antioxidant-enzyme activities and protein oxidation indicators differed between treatments.	[78]

DM, dry matter; PUFAs, polyunsaturated fatty acids; n-3, omega-3 fatty acids; n-6, omega-6 fatty acids. Dietary inclusion levels refer to the complete diet unless otherwise specified. Reported responses are specific to the feed ingredient, dietary formulation, livestock species and experimental conditions.

**Table 2:** Feed-resource strategies, benefits and implementation considerations for sustainable animal diets.

Feed-Resource Strategy	Representative Examples	Principal Benefits	Key Implementation Considerations
Forages and conserved fodders	Grazed pastures, cultivated fodder crops, hay, silages	Low-cost nutrients, soil cover, nutrient cycling, basis of ruminant productivity	Seasonality, drought and land degradation, conservation and grazing management
Leguminous forages and fodder trees	*Leucaena leucocephala*; *Gliricidia sepium*; *Desmodium* spp.; *Stylosanthes* spp.	Higher dietary protein, biological nitrogen fixation, reduced purchased protein	Species-specific harvest stage, anti-nutritional factors and safe inclusion rates
Silvopastoral systems	Integrated trees, shrubs, forages and grazing animals	Fodder and shade, animal welfare, nutrient cycling, carbon storage	Active management of light, pasture growth, animal class and grazing pressure
Crop residues and agro-industrial by-products	Cereal stovers; oilseed cakes; brewers’ and distillers’ grains, molasses, processing residues	Lower feed costs, fibre, energy, protein and minerals, improved circularity	Variable composition and digestibility, contamination risk, processing and testing
Biologically processed and alternative feeds	Fermented residues, microbial proteins, insect meals, algae and seaweeds	Improved nutrient availability in selected processed ingredients; diversification of feed supply; potential substitution of conventional feed resources	Process control, palatability, economics; life-cycle performance, regulatory approval

## Data Availability

No new data were created or analysed in this study. Data sharing is not applicable to this article.

## Supplementary Materials

The following supporting information can be downloaded at: https://www.mdpi.com/article/10.3390/ani16193138/s1. Table S1: Vaccine preventable diseases of livestock.

## Abbreviations

The following abbreviations are used in this manuscript:

CLA	Conjugated linoleic acid
HACCP	Hazard analysis and critical control point
MRLs	Maximum residue limits
MLs	Maximum levels
n-3	Omega-3 fatty acids
n-6	Omega-6 fatty acids
NIRS	Near-infrared spectroscopy
PSE	Pale, soft and exudative
PUFA	Polyunsaturated fatty acids
TMR	Total mixed ration

## References

[1] Bilotto F., Harrison M.T., Vibart R., Mackay A., Christie-Whitehead K.M., Ferreira C.S.S., Cottrell R.S., Forster D., Chang J. (2024). Towards resilient, inclusive, sustainable livestock farming systems. Trends Food Sci. Technol..

[2] Priyashantha H.P.H., Seresinhe T., Pathirana I.P.I., Gunawardana G.G., Silva G.L.L.P., Epa Arachchi A.E., Jayarathna S.J., Vidanarachchi J.K. (2025). Livestock and poultry production in Sri Lanka: Challenges and strategies for climate-resilient food security. Front. Sustain. Food Syst..

[3] FAO, UNEP, WHO, WOAH (2022). One Health Joint Plan of Action (2022–2026): Working Together for the Health of Humans, Animals, Plants and the Environment.

[4] Milazzo A., Liu J., Multani P., Steele S., Hoon E., Chaber A.-L. (2025). One Health implementation: A systematic scoping review using the Quadripartite One Health Joint Plan of Action. One Health.

[5] Ponnampalam E.N., Jairath G., Alves S.P., Gadzama I.U., Santhiravel S., Mapiye C., Holman B.W.B. (2025). Sustainable livestock production by utilising forages, supplements, and agricultural by-products: Enhancing productivity, muscle gain, and meat quality—A review. Meat Sci..

[6] Pinotti L., Cheli F., Govoni C., Rulli M.C., Premarajan P., Cattaneo D.M.I.R. (2025). The ‘One Nutrition’ approach: Connecting crop production, animal nutrition and human nutrition. Ital. J. Anim. Sci..

[7] McGrath J., Duval S.M., Tamassia L.F.M., Kindermann M., Stemmler R.T., de Gouvea V.N., Acedo T.S., Immig I., Williams S.N., Celi P. (2018). Nutritional strategies in ruminants: A lifetime approach. Res. Vet. Sci..

[8] Ponnampalam E.N., Kearns M., Kiani A., Santhiravel S., Vahmani P., Prache S., Monahan F.J., Mapiye C. (2024). Enrichment of ruminant meats with health enhancing fatty acids and antioxidants: Feed-based effects on nutritional value and human health aspects—Invited review. Front. Anim. Sci..

[9] Ponnampalam E.N., Holman B.W.B., Toldrá F. (2023). Sustainability II: Sustainable animal production and meat processing. Lawrie’s Meat Science.

[10] Ponnampalam E.N., Jairath G., Gadzama I.U., Li L., Santhiravel S., Ma C., Flores M., Priyashantha H. (2025). Production systems and feeding strategies in the aromatic fingerprinting of animal-derived foods: Invited review. Foods.

[11] Ponnampalam E.N., Priyashantha H., Vidanarachchi J.K., Kiani A., Holman B.W.B. (2024). Effects of nutritional factors on fat content, fatty acid composition, and sensorial properties of meat and milk from domesticated ruminants: An overview. Animals.

[12] Lauridsen C., Matte J.J., Lessard M., Celi P., Litta G. (2021). Role of vitamins for gastro-intestinal functionality and health of pigs. Anim. Feed Sci. Technol..

[13] Grace D. (2015). Food safety in low and middle income countries. Int. J. Environ. Res. Public Health.

[14] WOAH (2024). Animal Welfare: A Vital Asset for a More Sustainable World.

[15] Barkema H.W., von Keyserlingk M.A.G., Kastelic J.P., Lam T.J.G.M., Luby C., Roy J.-P., LeBlanc S.J., Keefe G.P., Kelton D.F. (2015). Invited review: Changes in the dairy industry affecting dairy cattle health and welfare. J. Dairy Sci..

[16] Grandin T. (2020). Livestock handling at the abattoir: Effects on welfare and meat quality. Meat Muscle Biol..

[17] Lara L.J., Rostagno M.H. (2013). Impact of heat stress on poultry production. Animals.

[18] Mashood Q., Karlsson A.H., Wallenbeck A., Eriksson S., Johansson A.M., Arvidsson Segerkvist K. (2024). A review on pale, soft, and exudative (PSE)-like zones in pork: Current knowledge on underlying factors and identification of knowledge gaps for further research. Acta Agric. Scand. Sect. A Anim. Sci..

[19] Makkar H.P.S. (2018). Review: Feed demand landscape and implications of food-not feed strategy for food security and climate change. Animal.

[20] Jose S., Dollinger J. (2019). Silvopasture: A sustainable livestock production system. Agrofor. Syst..

[21] Joseph J., Tramberend S., Kabi F., Fischer G., Kahil T. (2025). Sustainable intensification of fodder crop production can mitigate feed shortage and seasonality in East Africa. Environ. Dev..

[22] Amole T., Ayantunde A.A., Balehegn M., Adesogoan A.T. (2022). Livestock feed resources in the West African Sahel. Agron. J..

[23] Cooke A.S., Machekano H., Gwiriri L.C., Tinsley J.H.I., Silva G.M., Nyamukondiwa C., Safalaoh A., Morgan E.R., Lee M.R.F. (2025). The nutritional feed gap: Seasonal variations in ruminant nutrition and knowledge gaps in relation to food security in Southern Africa. Food Secur..

[24] Edwards A., Mlambo V., Mnisi C.M., Hughes M.P. (2024). Mature fruits of *Gliricidia sepium* and *Leucaena leucocephala* plants have potential as inexpensive protein and mineral supplements for ruminants. Agrofor. Syst..

[25] Tzec-Gamboa M., Álvarez-Rivera O.O., Solorio-Sánchez F.J. (2026). Contribution of biological nitrogen fixation to Leucaena forage production in intercropping systems with non-leguminous shrubs. Agrofor. Syst..

[26] Akpensuen T.T., Amodu J.T., Tanko R.J., Dele P.A., Bezerra L.R., Perazzo A.F. (2026). Harnessing African forage biodiversity through a hub-and-spoke model for sustainable livestock transformation. Anim. Front..

[27] Veldkamp T., Bosch G. (2015). Insects: A protein-rich feed ingredient in pig and poultry diets. Anim. Front..

[28] Parrini S., Aquilani C., Pugliese C., Bozzi R., Sirtori F. (2023). Soybean replacement by alternative protein sources in pig nutrition and its effect on meat quality. Animals.

[29] Dalle Zotte A., Singh Y., Pellattiero E., Palumbo B., Cullere M. (2024). Different lines of camelina (*Camelina sativa* (L.) Crantz) in broiler quails’ diets: Effects on meat physicochemical traits and sensory profile. Ital. J. Anim. Sci..

[30] Kokošková T., Pontalti E., Aremu T.S., Tůmová E., Chodová D., Bureš D., Lebedová N., Volek Z., Singh Y., Cullere M. (2025). Assessing the impact of dietary *Camelina sativa* (L.) Crantz inclusion on meat quality and sensory traits of growing rabbits. Meat Sci..

[31] Pomar C., Remus A. (2023). Review: Fundamentals, limitations and pitfalls on the development and application of precision nutrition techniques for precision livestock farming. Animal.

[32] Akintan O., Gebremedhin K.G., Uyeh D.D. (2024). Animal feed formulation—Connecting technologies to build a resilient and sustainable system. Animals.

[33] Antanaitis R., Džermeikaitė K., Bespalovaitė A., Ribelytė I., Rutkauskas A., Japertas S., Baumgartner W. (2023). Assessment of ruminating, eating, and locomotion behavior during heat stress in dairy cattle by using advanced technological monitoring. Animals.

[34] Martins L.F., Cueva S.F., Wasson D.E., Almeida C.V., Richards A.T., Stepanchenko N., Hristov A.N. (2025). Production and nutrient use efficiency of lactating dairy cows fed concentrate feeds via an automatic feeding system. J. Dairy Sci..

[35] Hossain M.E., Kabir M.A., Zheng L., Swain D.L., McGrath S., Medway J. (2024). Near-infrared spectroscopy for analysing livestock diet quality: A systematic review. Heliyon.

[36] Andretta I., Pomar C., Rivest J., Pomar J., Lovatto P.A., Radünz Neto J. (2014). The impact of feeding growing-finishing pigs with daily tailored diets using precision feeding techniques on animal performance, nutrient utilization, and body and carcass composition. J. Anim. Sci..

[37] Morey L., Bach A., Sabrià D., Riau V., Fernández B., Terré M. (2023). Effectiveness of precision feeding in reducing N excretion in dairy cattle. Anim. Feed Sci. Technol..

[38] Sandström V., Chrysafi A., Lamminen M., Troell M., Jalava M., Piipponen J., Siebert S., van Hal O., Virkki V., Kummu M. (2022). Food system by-products upcycled in livestock and aquaculture feeds can increase global food supply. Nat. Food.

[39] Shah A.M., Zhang H., Shahid M., Ghazal H., Shah A.R., Niaz M., Naz T., Ghimire K., Goswami N., Zhao H. (2025). The vital roles of agricultural crop residues and agro-industrial by-products to support sustainable livestock productivity in subtropical regions. Animals.

[40] Sun X., Dou Z., Shurson G.C., Hu B. (2024). Bioprocessing to upcycle agro-industrial and food wastes into high-nutritional value animal feed for sustainable food and agriculture systems. Resour. Conserv. Recycl..

[41] Deng J., Huang J.-C., Xu Z.-J., Liu Y., Karrow N.A., Liu M., Sun L.H. (2023). Remediation strategies for mycotoxins in animal feed. Toxins.

[42] (2004). Code of Practice on Good Animal Feeding.

[43] Santos G.T., Lima L.S., Schogor A.L.B., Romero J.V., De Marchi F.E., Grande P.A., Santos N.W., Santos F.S., Kazama R. (2014). Citrus pulp as a dietary source of antioxidants for lactating Holstein cows fed highly polyunsaturated fatty acid diets. Asian-Australas. J. Anim. Sci..

[44] Chedea V.S., Pelmus R.S., Lazar C., Pistol G.C., Calin L.G., Toma S.M., Dragomir C., Taranu I. (2017). Effects of a diet containing dried grape pomace on blood metabolites and milk composition of dairy cows. J. Sci. Food Agric..

[45] Steyn L., Meeske R., Cruywagen C.W. (2018). The effect of replacing maize with dried apple pomace in the concentrate on performance of Jersey cows grazing kikuyu pasture. Anim. Feed Sci. Technol..

[46] Castellani F., Vitali A., Bernardi N., Marone E., Palazzo F., Grotta L., Martino G. (2017). Dietary supplementation with dried olive pomace in dairy cows modifies the composition of fatty acids and the aromatic profile in milk and related cheese. J. Dairy Sci..

[47] Oliveira R.L., Neto S.G., de Lima F.H.S., de Medeiros A.N., Bezerra L.R., Pereira E.S., Bagaldo A.R., de Pellegrini C.B., Correia B.R. (2016). Composition and fatty acid profile of milk from cows supplemented with pressed oilseed cake. Anim. Sci. J..

[48] Rutkowska J., Białek M., Bagnicka E., Jarczak J., Tambor K., Strzałkowska N., Jóźwik A., Krzyżewski J., Adamska A., Rutkowska E. (2015). Effects of replacing extracted soybean meal with rapeseed cake in corn grass silage-based diet for dairy cows. J. Dairy Res..

[49] Oancea A.G., Dragomir C., Cișmileanu A. (2022). The effects of minor oilseeds cakes on rumen metabolism and productive performances of ruminants. Arch. Zootech..

[50] Karlsson L., Finell M., Martinsson K. (2010). Effects of increasing amounts of hempseed cake in the diet of dairy cows on the production and composition of milk. Animal.

[51] Seneviratne R.W., Beltranena E., Newkirk R.W., Goonewardene L.A., Zijlstra R.T. (2011). Processing conditions affect nutrient digestibility of cold-pressed canola cake for grower pigs. J. Anim. Sci..

[52] Almeida F.N., Htoo J.K., Thomson J., Stein H.H. (2013). Amino acid digestibility in camelina products fed to growing pigs. Can. J. Anim. Sci..

[53] Kahindi R.K., Woyengo T.A., Thacker P.A., Nyachoti C.M. (2014). Energy and amino acid digestibility of camelina cake fed to growing pigs. Anim. Feed Sci. Technol..

[54] Presto M.H., Lyberg K., Lindberg J.E. (2011). Digestibility of amino acids in organically cultivated white-flowering faba bean and cake from cold-pressed rapeseed, linseed and hemp seed in growing pigs. Arch. Anim. Nutr..

[55] Kim J.W., Koo B., Nyachoti C.M. (2017). Digestible, metabolizable, and net energy of camelina cake fed to growing pigs and additivity of energy in mixed diets. J. Anim. Sci..

[56] Taranu I., Gras M., Pistol G.C., Motiu M., Marin D.E., Lefter N., Ropota M., Habeanu M. (2014). ω-3 PUFA-rich Camelina oil by-products improve the systemic metabolism and spleen cell functions in fattening pigs. PLoS ONE.

[57] Kaczmarek P., Korniewicz D., Lipiński K., Mazur-Kuśnirek M. (2019). The effect of hydrothermally processed soybean- and rapeseed-based diets on performance, meat and carcass quality characteristics in growing-finishing pigs. Ann. Anim. Sci..

[58] Silversides F.G., Lefrançois M.R. (2005). The effect of feeding hemp seed meal to laying hens. Br. Poult. Sci..

[59] Halle I., Schöne F. (2013). Influence of rapeseed cake, linseed cake and hemp seed cake on laying performance of hens and fatty acid composition of egg yolk. J. Verbrauch. Leb..

[60] Orczewska-Dudek S., Pietras M. (2019). The effect of dietary *Camelina sativa* oil or cake in the diets of broiler chickens on growth performance, fatty acid profile, and sensory quality of meat. Animals.

[61] Untea A.E., Panaite T.D., Dragomir C., Ropota M., Olteanu M., Varzaru I. (2019). Effect of dietary chromium supplementation on meat nutritional quality and antioxidant status from broilers fed with Camelina-meal-supplemented diets. Animal.

[62] Orczewska-Dudek S., Pietras M., Puchała M., Nowak J. (2020). *Camelina sativa* oil and camelina cake as sources of polyunsaturated fatty acids in the diets of laying hens: Effect on hen performance, fatty acid profile of yolk lipids, and egg sensory quality. Ann. Anim. Sci..

[63] Cullere M., Singh Y., Pellattiero E., Berzuini S., Galasso I., Clemente C., Dalle Zotte A. (2023). Effect of the dietary inclusion of *Camelina sativa* cake into quail diet on live performance, carcass traits, and meat quality. Poult. Sci..

[64] Dalle Zotte A., Palumbo B., Pellattiero E., Singh Y., Zanetti F., Cullere M. (2026). Effects of dietary camelina (*Camelina sativa*) cake from different lines on digestibility, feed preference, performance, and egg quality in laying quail. Poult. Sci..

[65] Baláži A., Svoradová A., Kováčik A., Vašíček J., Chrenek P. (2024). The effects of adding hempseed cake on sperm traits, body weight, haematological and biochemical parameters in rabbit males. Vet. Sci..

[66] Volek Z., Šufliarský P., Taubner T., Plachý V., Kokošková T., Aremu T., Cullere M., Galasso I., Chodová D., Tůmová E. (2025). Effect of replacing rapeseed cake with camelina (*Camelina sativa* (L.) Crantz) cake on nutrient digestibility, pancreatic enzyme activity, growth performance and carcass traits in fattening rabbits. Anim. Feed Sci. Technol..

[67] Cullere M., Tasoniero G., Giaccone V., Miotti-Scapin R., Claeys E., De Smet S., Dalle Zotte A. (2016). Black soldier fly as dietary protein source for broiler quails: Apparent digestibility, excreta microbial load, feed choice, performance, carcass and meat traits. Animal.

[68] Elahi U., Wang J., Ma Y., Wu J., Qi G., Zhang H. (2020). Evaluation of yellow mealworm meal as a protein feedstuff in the diet of broiler chicks. Animals.

[69] Sedgh-Gooya S., Torki M., Darbemamieh M., Khamisabadi H., Abdolmohamadi A. (2022). Growth performance and intestinal morphometric features of broiler chickens fed on dietary inclusion of yellow mealworm (*Tenebrio molitor*) larvae powder. Vet. Med. Sci..

[70] Baderuddin S.H., David L.S., Wester T.J., Morel P.C.H. (2024). Influence of different levels of black soldier fly larvae meal on growth performance and carcass quality of broiler chickens. Livest. Sci..

[71] Saidani M., Dabbou S., Larbi M.B., Silmen I.B., Fraihi W., Arbi T., Amraoui M., M’Hamdi N. (2025). Effect of black soldier fly (*Hermetia illucens*) larvae meal on growth performance, carcass characteristics, meat quality, and cecal microbiota in broiler chickens. Front. Anim. Sci..

[72] El Basuini M.F., Khattab A.A.A., Hafsa S.H.A., Teiba I.I., Elkassas N.E.M., El-Bilawy E.H., Dawood M.A.O., Atia S.E.S. (2023). Impacts of algae supplements (*Arthrospira* & *Chlorella*) on growth, nutrient variables, intestinal efficacy, and antioxidants in New Zealand white rabbits. Sci. Rep..

[73] Abdou A.A., Raafat S.M., Younan G.E., Farag M.E., Abd El-Wahab W.M., Shahin G.F., El-Naharwy M.M., Khattab A.R. (2024). Effect of Spirulina algae levels supplements on the productive and reproductive performance of doe rabbits. Egypt. J. Vet. Sci..

[74] Dalle Zotte A., Cullere M., Sartori A., Dal Bosco A., Gerencsér Z., Matics Z., Kovács M., Szendrő Z. (2014). Effect of dietary supplementation of spirulina (*Arthrospira platensis*) and thyme (*Thymus vulgaris*) on carcass composition, meat physical traits, and vitamin B12 content on growing rabbits. World Rabbit Sci..

[75] da Silva G.G., Ferreira de Jesus E., Takiya C.S., Del Valle T.A., da Silva T.H., Vendramini T.H.A., Yu E.J., Rennó F.P. (2016). Short communication: Partial replacement of ground corn with algae meal in a dairy cow diet: Milk yield and composition, nutrient digestibility, and metabolic profile. J. Dairy Sci..

[76] Lamminen M., Halmemies-Beauchet-Filleau A., Kokkonen T., Simpura I., Jaakkola S., Vanhatalo A. (2017). Comparison of microalgae and rapeseed meal as supplementary protein in the grass silage-based nutrition of dairy cows. Anim. Feed Sci. Technol..

[77] Kulpys J., Paulauskas E., Pilipavičius V., Stankevičius R. (2009). Influence of cyanobacteria *Arthrospira* (*Spirulina*) *platensis* biomass additives towards the body condition of lactation cows and biochemical milk indexes. Agron. Res..

[78] Tsiplakou E., Abdullah M.A.M., Mavrommatis A., Chatzikonstantinou M., Skliros D., Sotirakoglou K., Flemetakis E., Labrou N.E., Zervas G. (2018). The effect of dietary *Chlorella vulgaris* inclusion on goat’s milk chemical composition, fatty acids profile and enzymes activities related to oxidation. J. Anim. Physiol. Anim. Nutr..

[79] Serrapica F., Lambiase C., Masucci F., Braghieri A., Lorenzo J.M., Di Francia A. (2025). Feed-grade hempseed as alternative protein source in organic broilers diet: Growth performance, carcass characteristics and meat quality. J. Agric. Food Res..

[80] Masucci F., Scalera G., Serrapica F., Braghieri A., Sabia E., Riviezzi A.M., De Luca L., Romano R., De Rosa G., Lambiase C. (2026). Integrating hemp cake in organic layer diets: A comprehensive assessment of egg quality, hen behavior, production costs, and life cycle impact. J. Agric. Food Res..

[81] Yafetto L., Odamtten G.T., Wiafe-Kwagyan M. (2023). Valorization of agro-industrial wastes into animal feed through microbial fermentation: A review of the global and Ghanaian case. Heliyon.

[82] Jairath G., Verma A.K., Rani D., Marappan G., Subbanna Y.B., Singh B., Mal G., Gopinath D., Sharma R., Katoch S. (2023). Self-fermented agro-wastes as antioxidant enriched maize grain replacer for sustainable animal feeding. J. Clean. Prod..

[83] Chisoro P.C., Jaja I.F., Assan N. (2023). Incorporation of local novel feed resources in livestock feed for sustainable food security and circular economy in Africa. Front. Sustain..

[84] Thornton P., Gurney-Smith H., Wollenberg E. (2023). Alternative sources of protein for food and feed. Curr. Opin. Environ. Sustain..

[85] (1995). General Standard for Contaminants and Toxins in Food and Feed.

[86] Tolosa J., Rodríguez-Carrasco Y., Ruiz M.J., Vila-Donat P. (2021). Multi-mycotoxin occurrence in feed, metabolism and carry-over to animal-derived food products: A review. Food Chem. Toxicol..

[87] Codex Alimentarius Commission (2003). Working Principles for Risk Analysis for Application in the Framework of the Codex Alimentarius. Annex 1: Definitions.

[88] FAO, IFIF (2020). Good Practices for the Feed Sector: Implementing the Codex Alimentarius Code of Practice on Good Animal Feeding.

[89] Suttle N.F. (2010). Mineral Nutrition of Livestock.

[90] Lauridsen C., Matte J.J., Wiseman J. (2017). Recent advances in understanding the role of vitamins in pig nutrition. Achieving Sustainable Production of Pig Meat: Animal Breeding and Nutrition.

[91] Calder P.C. (2020). Nutrition, immunity and COVID-19. BMJ Nutr. Prev. Health.

[92] Kobek-Kjeldager C., Schönherz A.A., Canibe N., Pedersen L.J. (2022). Diet and microbiota-gut-brain axis in relation to tail biting in pigs: A review. Appl. Anim. Behav. Sci..

[93] Jadhav V.V., Han J., Fasina Y., Harrison S.H. (2022). Connecting gut microbiomes and short chain fatty acids with the serotonergic system and behavior in *Gallus gallus* and other avian species. Front. Physiol..

[94] Goff J.P. (2008). The monitoring, prevention, and treatment of milk fever and subclinical hypocalcemia in dairy cows. Vet. J..

[95] Plaizier J.C., Krause D.O., Gozho G.N., McBride B.W. (2008). Subacute ruminal acidosis in dairy cows: The physiological causes, incidence and consequences. Vet. J..

[96] Hu R., Li S., Diao H., Huang C., Yan J., Wei X., Zhou M., He P., Wang T., Fu H. (2023). The interaction between dietary fiber and gut microbiota, and its effect on pig intestinal health. Front. Immunol..

[97] Bedford M.R., Svihus B., Cowieson A.J. (2024). Dietary fibre effects and the interplay with exogenous carbohydrases in poultry nutrition. Anim. Nutr..

[98] Thornton P.K. (2010). Livestock production: Recent trends, future prospects. Philos. Trans. R. Soc. B Biol. Sci..

[99] Lauridsen C., Højberg O., Canibe N., Bailey M., Stokes C. (2022). Improving gut function in pigs to prevent dysbiosis and post-weaning diarrhoea. Understanding Gut Microbiomes as Targets for Improving Pig Gut Health.

[100] Lauridsen C., Smidt H., Fredholm M., Marco M.L., Arumugam M. (2026). Reducing antimicrobial resistance burden from livestock production by targeting pig gut health. Front. Vet. Sci..

[101] Callaway T.R., Lillehoj H., Chuanchuen R., Gay C.G. (2021). Alternatives to antibiotics: A symposium on the challenges and solutions for animal production. Antibiotics.

[102] Canibe N., Højberg O., Kongsted H., Vodolazska D., Lauridsen C., Nielsen T.S., Schönherz A.A. (2022). Review on preventive measures to reduce post-weaning diarrhoea in piglets. Animals.

[103] LeBlanc S.J., Lissemore K.D., Kelton D.F., Duffield T.F., Leslie K.E. (2006). Major advances in disease prevention in dairy cattle. J. Dairy Sci..

[104] WOAH (2024). Chapter 6.10. Responsible and Prudent Use of Antimicrobial Agents in Veterinary Medicine. Terrestrial Animal Health Code.

[105] Alders R.G., Campbell A., Costa R., Guèye E.F., Hoque M.A., Perezgrovas-Garza R., Rota A., Wingett K. (2021). Livestock across the world: Diverse animal species with complex roles in human societies and ecosystem services. Anim. Front..

[106] Hoelzer K., Bielke L., Blake D.P., Cox E., Cutting S.M., Devriendt B., Erlacher-Vindel E., Goossens E., Karaca K., Lemiere S. (2018). Vaccines as alternatives to antibiotics for food producing animals. Part 1: Challenges and needs. Vet. Res..

[107] Plowright R.K., Reaser J.K., Locke H., Woodley S.J., Patz J.A., Becker D.J., Oppler G., Hudson P.J., Tabor G.M. (2021). Land use-induced spillover: A call to action to safeguard environmental, animal, and human health. Lancet Planet. Health.

[108] EFSA, ECDC (2025). The European Union summary report on antimicrobial resistance in zoonotic and indicator bacteria from humans, animals and food in 2022–2023. EFSA J..

[109] Broom D.M., Fraser A.F. (2015). Domestic Animal Behaviour and Welfare.

[110] Oluwagbenga E.M., Fraley G.S. (2023). Heat stress and poultry production: A comprehensive review. Poult. Sci..

[111] Apalowo O.O., Ekunseitan D.A., Fasina Y.O. (2024). Impact of heat stress on broiler chicken production. Poultry.

[112] Cartoni Mancinelli A., Baldi G., Soglia F., Mattioli S., Sirri F., Petracci M., Castellini C., Zampiga M. (2023). Impact of chronic heat stress on behavior, oxidative status and meat quality traits of fast-growing broiler chickens. Front. Physiol..

[113] Ross J.W., Hale B.J., Gabler N.K., Rhoads R.P., Keating A.F., Baumgard L.H. (2015). Physiological consequences of heat stress in pigs. Anim. Prod. Sci..

[114] Gourdine J.L., Rauw W.M., Gilbert H., Poullet N. (2021). The genetics of thermoregulation in pigs: A review. Front. Vet. Sci..

[115] Liu F., Zhao W., Le H.H., Cottrell J.J., Green M.P., Leury B.J., Dunshea F.R., Bell A.W. (2022). What have we learned about the effects of heat stress on the pig industry?. Animal.

[116] Marai I.F.M., Habeeb A.A.M., Gad E.A. (2002). Rabbits’ productive, reproductive and physiological performance traits as affected by heat stress: A review. Livest. Prod. Sci..

[117] Pontalti E., Matics Z., Cullere M., Szendrő Z., Gerencsér Z., Palumbo B., Dalle Zotte A. (2025). Growth performance, carcass and meat quality traits of three rabbit lines under heat stress conditions. Animals.

[118] de Jong I.C., Blaauw X.E., van der Eijk J.A.J., da Silva C.S., van Krimpen M., Molenaar R., van der Brand H. (2021). Providing environmental enrichments affects activity and performance, but not leg health in fast- and slower-growing broiler chickens. Appl. Anim. Behav. Sci..

[119] Meyer M.M., Johnson A.K., Bobeck E.A. (2023). Breast muscle white striping and serum corticosterone reduced in broilers exposed to laser environmental enrichment. Poult. Sci..

[120] Nazareno A.C., Silveira R.M.F., Fernandes D.P.B., Chierri J., Pradella L.O., da Silva I.J.O. (2024). Perches used as environmental enrichment influence fast-growth broilers’ biomechanics and locomotor morphometry at the age of 42 days. PLoS ONE.

[121] Grandin T. (2021). The visual, auditory, and physical environment of livestock handling facilities and its effect on animal movement and welfare. Front. Anim. Sci..

[122] Bishop S.C., Woolliams J.A. (2014). Genomics and disease resistance studies in livestock. Livest. Sci..

[123] Kasimanickam R., Kasimanickam V., Moore D.A., Kastelic J.P. (2025). Application of genomic selection in beef cattle disease resistance: A review. Animals.

[124] Salim B., Alasmari S., Bakheit M.A. (2025). Genetic resistance to vector-borne hemoprotozoa in livestock: Molecular markers, host-parasite interactions, and implications for breeding and control. J. Vet. Sci..

[125] Menchetti L., Birolo M., Mugnai C., Mancinelli A.C., Xiccato G., Trocino A., Castellini C. (2024). Effect of genotype and nutritional and environmental challenges on growth curve dynamics of broiler chickens. Poult. Sci..

[126] Rauw W.M., Baumgard L.H., Dekkers J.C.M. (2025). Review: Feed efficiency and metabolic flexibility in livestock. Animal.

[127] Petracci M., Soglia F., Madruga M., Carvalho L., Ida E., Estévez M. (2019). Wooden-breast, white striping, and spaghetti meat: Causes, consequences and consumer perception of emerging broiler meat abnormalities. Compr. Rev. Food Sci. Food Saf..

[128] Baldi G., Soglia F., Petracci M. (2021). Spaghetti meat abnormality in broilers: Current understanding and future research directions. Front. Physiol..

[129] World Health Organization (2026). Antimicrobial Resistance. https://www.who.int/news-room/fact-sheets/detail/antimicrobial-resistance.

[130] Velazquez-Meza M.E., Galarde-López M., Carrillo-Quiróz B., Alpuche-Aranda C.M. (2022). Antimicrobial resistance: One Health approach. Vet. World.

[131] Pandey S., Doo H., Keum G.B., Kim E.S., Kwak J., Ryu S., Choi Y., Kang J., Kim S., Lee N.R. (2024). Antibiotic resistance in livestock, environment and humans: One Health perspective. J. Anim. Sci. Technol..

[132] GBD 2021 Antimicrobial Resistance Collaborators (2024). Global burden of bacterial antimicrobial resistance 1990–2021: A systematic analysis with forecasts to 2050. Lancet.

[133] Beal T., Gardner C.D., Herrero M., Iannotti L.L., Merbold L., Nordhagen S., Mottet A. (2023). Friend or foe? The role of animal-source foods in healthy and environmentally sustainable diets. J. Nutr..

[134] Paro F.R., Davour R., Acosta D., Mechlowitz K., Tiwari C., McKune S.L. (2025). Improving nutrition security in low- and middle-income countries and the role of animal-source foods. Annu. Rev. Anim. Biosci..

[135] Bagdas R., Jairath G., Varun, Bishnoi S., Subbanna Y.B., Mal G., Biswas A.K., Dutt T., Sen A.R., Bhatt R.S. (2026). Replacing maize grain with self-fermented agro-waste in total mixed ration improves fatty acid profile, oxidative stability and meat quality traits in goats. Meat Sci..

[136] World Health Organization (2026). Food Safety. https://www.who.int/news-room/fact-sheets/detail/food-safety.

[137] (2023). General Principles of Food Hygiene.

[138] Zentai A., Jóźwiak Á., Süth M., Farkas Z. (2023). Carry-over of aflatoxin B1 from feed to cow milk: A review. Toxins.

[139] EFSA CONTAM Panel (2026). Update of the risk assessment on dioxins and dioxin-like PCBs in feed and food. EFSA J..

[140] European Food Safety Authority (2025). Veterinary Drug Residues in Food—Non-Compliance Stays Low in 2023. https://www.efsa.europa.eu/en/news/veterinary-drug-residues-food-non-compliance-stays-low-2023.

[141] Rana M.S., Lee S.Y., Kang H.J., Hur S.J. (2019). Reducing veterinary drug residues in animal products: A review. Food Sci. Anim. Resour..

[142] Codex Alimentarius Commission (2026). Definitions for the Purposes of the Codex Alimentarius. Codex Alimentarius Procedural Manual.

[143] Kazemi M., Valizadeh R. (2026). Nutritional strategies for enhancing the quality of animal-derived food products. Discov. Food.

[144] FAO (2018). World Livestock: Transforming the Livestock Sector Through the Sustainable Development Goals.

[145] Herrero M., Havlík P., Valin H., Notenbaert A., Rufino M.C., Thornton P.K., Blümmel M., Weiss F., Grace D., Obersteiner M. (2013). The roles of livestock in developing countries. Animal.

[146] Schneider K.R., Fanzo J., Haddad L., Herrero M., Rosero Moncayo J., Herforth A., Remans R., Guarin A., Resnick D., Covic N. (2023). The state of food systems worldwide in the countdown to 2030. Nat. Food.

[147] Banda L.J., Tanganyika J., Koczberski G. (2021). Livestock provide more than food in smallholder production systems of developing countries. Anim. Front..

[148] Collishaw A., Janzen S., Mullally C., Camilli H. (2023). A review of livestock development interventions’ impacts on household welfare in low- and middle-income countries. Glob. Food Secur..

[149] Bora M., Goswami R.N., Das D., Zaman G., Das A., Nath M. (2010). Combining ability effects on post weaning growth performance in terms of average daily gain in body weight in rabbit. Tamilnadu J. Vet. Anim. Sci..

[150] Cullere M., Dalle Zotte A. (2018). Rabbit meat production and consumption: State of knowledge and future perspectives. Meat Sci..

[151] FAO (2019). Developing Sustainable Value Chains for Small-Scale Livestock Producers.

[152] Mottet A., Tempio G. (2017). Global poultry production: Current state and future outlook and challenges. World’s Poult. Sci. J..

[153] Quisumbing A., Sproule K., Martinez E.M., Malapit H.J. (2023). Measuring women’s empowerment in agriculture. Glob. Food Secur..

[154] Hira F.T.Z., Alam M.J., Begum I.A. (2025). Women’s empowerment in livestock sector as a tool to enhance child’s nutrition: A review. Discov. Sustain..

[155] Garcia S.N., Osburn B.I., Jay-Russell M.T. (2020). One Health for food safety, food security, and sustainable food production. Front. Sustain. Food Syst..

[156] Nguyen-Viet H., Lâm S., Alonso S., Unger F., Moodley A., Bett B., Fèvre E.M., Knight-Jones T.J.D., Mor S.M., Nguyen H.T.T. (2025). Insights and future directions: Applying the One Health approach in international agricultural research for development to address food systems challenges. One Health.

[157] FAO (2025). The Role of Livestock in Circular Bioeconomy Systems.

[158] McAllister T.A., Becquet P., Amon B.R., Lee M.R.F., LEAP TAG (2025). Role of livestock in circular bioeconomy systems. Anim. Front..

[159] van Huis A., Rumbos C.I., Oonincx D.G.A.B., Rojo S., Kovitvadhi A., Gasco L. (2024). From traditional to industrial use of insects as feed: A review. J. Insects Food Feed.

[160] Oonincx D.G.A.B., van Itterbeeck J., Heetkamp M.J.W., van den Brand H., van Loon J.J.A., van Huis A. (2010). An exploration on greenhouse gas and ammonia production by insect species suitable for animal or human consumption. PLoS ONE.

[161] van Huis A., Oonincx D.G.A.B. (2017). The environmental sustainability of insects as food and feed. A review. Agron. Sustain. Dev..

[162] Amorim H.C., Ashworth A.J., Arsi K., Rojas M.G., Morales-Ramos J.A., Donoghue A., Robinson K. (2024). Insect frass composition and potential use as an organic fertilizer in circular economies. J. Econ. Entomol..

[163] Lomonaco G., Franco A., De Smet J., Scieuzo C., Salvia R., Falabella P. (2024). Larval frass of *Hermetia illucens* as organic fertilizer: Composition and beneficial effects on different crops. Insects.

[164] Tóth M., Birolo M., Palumbo B., Nalon E., Frangipane di Regalbono A., Giaccone V., Erdélyi M., Dalle Zotte A. (2026). Effect of insect farming by-product (frass) as a novel bedding material on litter quality, growth performance, carcass traits, and meat quality of broiler chickens. Front. Anim. Sci..

[165] Sadeghpour A., Keshavarz Afshar R. (2024). Livestock manure: From waste to resource in a circular economy. J. Agric. Food Res..

[166] Gerber P.J., Steinfeld H., Henderson B., Mottet A., Opio C., Dijkman J., Falcucci A., Tempio G. (2013). Tackling Climate Change Through Livestock: A Global Assessment of Emissions and Mitigation Opportunities.

[167] FAO (2023). Pathways Towards Lower Emissions: A Global Assessment of the Greenhouse Gas Emissions and Mitigation Options from Livestock Agrifood Systems.

[168] Herrero M., Thornton P.K., Gerber P., Reid R.S. (2009). Livestock, livelihoods and the environment: Understanding the trade-offs. Curr. Opin. Environ. Sustain..

