# Supplementary material for: Balanced Animal Nutrition for Better Feeds, Healthy Animals, Safe Food, and a Healthy Planet: A One Health Perspective—An Invited Review
Source: Animals (Basel). 2026 Oct 7;16(19):3138. doi: 10.3390/ani16193138 (PMC13663420; doi:10.3390/ani16193138)
Supplement: Supplementary file 1 [file animals-16-03138-s001.zip › animals-4568587-supplementary.pdf]

animals

MDPI

Table S1. Vaccine preventable diseases of livestock.

Category of Pathogen	Genome type	Vaccinable livestock diseases	Main livestock affected
Bacterial	DNA	Anthrax (*Bacillus anthracis*)	Cattle, sheep, goats, pigs
Bacterial	DNA	Blackleg/black quarter (*Clostridium chauvoei*)	Cattle, sheep
Bacterial	DNA	Enterotoxemia (*Clostridium perfringens*)	Sheep, goats, cattle
Bacterial	DNA	Tetanus (*Clostridium tetani*)	Horses, sheep, goats, cattle, pigs
Bacterial	DNA	Brucellosis (*Brucella abortus, B. melitensis, B. suis*)	Cattle, sheep, goats, pigs
Bacterial	DNA	Leptospirosis (*Leptospira* spp.)	Cattle, pigs, sheep, goats
Bacterial	DNA	Tuberculosis (*Mycobacterium bovis*)	Cattle and other ruminants; vaccination is limited or program-dependent
Bacterial	DNA	Pasteurellosis/respiratory disease (*Mannheimia haemolytica, Pasteurella multocida*)	Cattle, sheep, goats
Bacterial	DNA	Contagious caprine pleuropneumonia (*Mycoplasma capricolum* subsp. *capripneumoniae*)	Goats
Bacterial	DNA	Enzootic abortion of ewes (*Chlamydia abortus*)	Sheep, goats
Bacterial	DNA	Anthrax (*Bacillus anthracis*)	Cattle, sheep, goats, pigs
Bacterial	DNA	Blackleg/black quarter (*Clostridium chauvoei*)	Cattle, sheep
Bacterial	DNA	Swine erysipelas (Erysipelothrix rhusiopathiae)	Pigs
Bacterial	DNA	Fowl cholera (Pasteurella multocida)	Poultry
Bacterial	DNA	Infectious coryza (Avibacterium paragallinarum)	Chickens
Bacterial	DNA	Mycoplasmosis, including Mycoplasma gallisepticum disease	Poultry
Viral	DNA virus	Lumpy skin disease (Capripoxvirus)	Cattle, buffalo
Viral	DNA virus	Sheep pox and goat pox (Capripoxvirus)	Sheep, goats
Viral	DNA virus	African swine fever (Asfarvirus)	Pigs; vaccines are not universally available and use is jurisdiction-dependent
Viral	DNA virus	Infectious bovine rhinotracheitis—IBR (Bovine herpesvirus 1)	Cattle

Viral	DNA virus	Aujeszky's disease/pseudorabies (Suid alphaherpesvirus 1)	Pigs
Viral	DNA virus	Fowl pox (Avipoxvirus)	Chickens, turkeys
Viral	DNA virus	Marek's disease (Marek's disease virus)	Chickens
Viral	DNA virus	Infectious laryngotracheitis (Gallid alphaherpesvirus 1)	Chickens
Viral	RNA virus	Foot-and-mouth disease (Aphthovirus)	Cattle, sheep, goats, pigs
Viral	RNA virus	Rabies (Lyssavirus)	All mammals, including livestock
Viral	RNA virus	Peste des petits ruminants—PPR (Morbillivirus)	Sheep, goats
Viral	RNA virus	Rift Valley fever (Phlebovirus)	Cattle, sheep, goats, camels
Viral	RNA virus	Bluetongue (Orbivirus)	Sheep, cattle, goats
Viral	RNA virus	Bovine viral diarrhea—BVD (Pestivirus)	Cattle
Viral	RNA virus	Porcine reproductive and respiratory syndrome—PRRS (Arterivirus)	Pigs
Viral	RNA virus	Newcastle disease (Avian orthoavulavirus 1)	Chickens and other poultry
Viral	RNA virus	Avian influenza (Influenzavirus A)	Poultry; vaccination is regulated and strain-dependent
Viral	RNA virus	Infectious bronchitis (Gammacoronavirus)	Chickens
Viral	RNA virus	Rotavirus diarrhea	Calves, lambs, piglets
Viral	RNA virus	Border disease (Pestivirus)	Sheep and goats
Viral	RNA virus	Equine influenza (Influenzavirus A)	Horses
Viral	RNA virus	Equine viral arteritis (Arterivirus)	Horses
Viral	RNA virus	Porcine reproductive and respiratory syndrome—PRRS (Arterivirus)	Pigs

Note: All bacteria have DNA genomes. The "DNA vs RNA" distinction is therefore most useful for viral diseases. However, whether a disease is vaccinated against depends on country, vaccine licensing, circulating strains, animal species, and disease-control policy.

https://doi.org/10.3390/ani16193138
